# Natural immune escape mutations in the envelope proteins of hepatitis B virus (HBV) regulate the assembly and infectivity of human hepatitis delta virus (HDV)

**DOI:** 10.1128/jvi.01063-26

**Published:** 2026-09-01

**Authors:** Oleksandra Chazova, Igor Zaiets, Severin O. Gudima

**Affiliations:** 1 Department of Microbiology, Molecular Genetics and Immunology, University of Kansas Medical Center 21638, Kansas City, Kansas, USA; Wake Forest University School of Medicine, Winston-Salem, North Carolina, USA

**Keywords:** anti-HBV immune response, HBV immune escape mutants, natural mutations in HBsAg, major antigenic loop, assembly and infectivity of HDV

## Abstract

The study examined how 35 natural immune escape mutations (IEMs) of hepatitis B virus (HBV) located in the major antigenic loop (MAL) of the envelope proteins regulate the assembly and infectivity of the natural sub-viral agent of HBV, hepatitis delta virus (HDV). All IEMs were permissive for the assembly and secretion of HDV virions. Only seven IEMs resulted in considerably decreased levels of secreted HDV virions in the context of one to three tested HBV genotypes, B, C, or D. The effects of IEMs on HDV infectivity were much more pronounced than on the assembly. Thirteen IEMs considerably inhibited the infectivity of HDV virions. All of them greatly reduced both the levels of HDV RNA genomes accumulated in the infected cells and the percentage of HDV-infected cells. Only one mutant R(169)P generated completely non-infectious HDV virions. Our analysis of the IEMs-mediated effects on the HDV life cycle therefore identified a number of critically important amino acid residues in the MAL, and aided the mechanistic understanding of the MAL functioning during the processes of HDV assembly and infectivity. The inhibition of either HDV assembly and/or infectivity is expected to suppress the viral spread and super-infection *in vivo*, and therefore to reduce the HDV reservoir in infected livers. Overall, the generated data showed that the anti-HBV immune response is capable of considerable down-regulation of the concomitant HDV infection by facilitating the selection for IEMs in the MAL. The study further advanced our understanding of the mechanisms of the complex HBV-HDV and HDV-host interactions.

**IMPORTANCE:**

The study demonstrated that anti-hepatitis B virus (HBV) immune response via selection for the immune escape mutations (IEMs) in HBV envelope proteins (surface antigen, HBsAg) can considerably reduce the assembly and/or infectivity of hepatitis delta virus (HDV) virions coated with HBsAg. The IEMs could down-regulate concomitant HDV infection mostly via affecting HDV infectivity, which was considerably inhibited by 13 out of 35 IEMs. The HDV spread/super-infection, which are expected to be suppressed by inhibited via HBV-specific IEMs the assembly and/or infectivity of HDV virions, should result in a reduced HDV reservoir in infected livers. The examination of the inhibition of HDV infectivity by IEMs apparently indicated that the major antigenic loop (MAL) of HBsAg could contain the residues primarily involved in viral attachment/entry and also the residues mostly regulating intracellular post-entry trafficking of HDV virions. The data advance our understanding of the MAL functioning during infection and of the indirect regulation of the HDV life cycle through the anti-HBV immune response.

## INTRODUCTION

**Fig 1:**
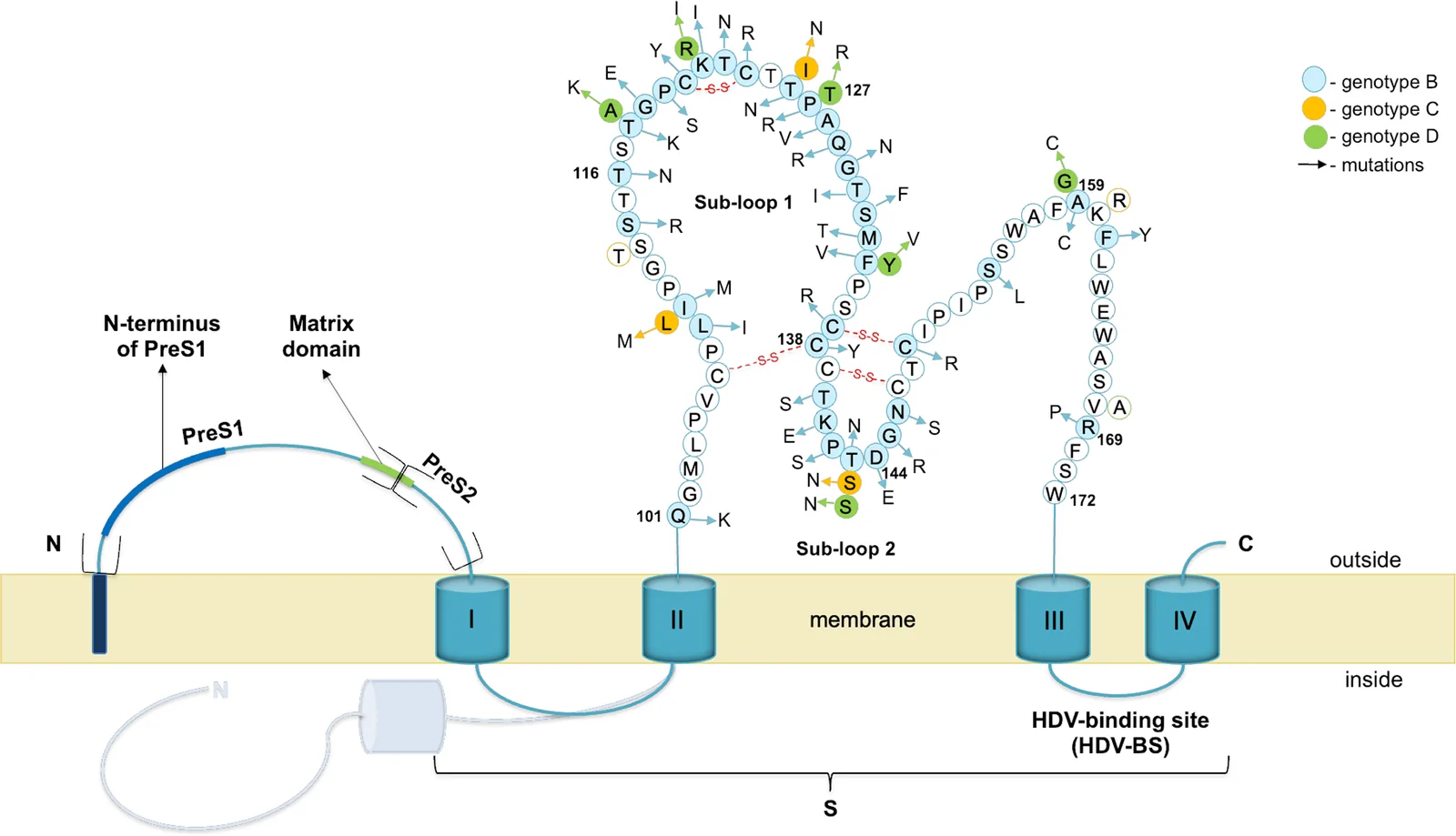
The structure of the large envelope protein of HBV. The figure depicts the structure of the large envelope protein (L) embedded into the membrane of the virion. The inside and outside spaces are indicated. Shown are the PreS1, PreS2, and S domains of the L. Also shown are four transmembrane domains (TMDs), the MAL with internal S–S bonds and formed sub-loops on the outside between the second (II) and third (III) TMDs, and the HDV-binding site (HDV-BS) that is a small cytosolic loop on the inside between the third (III) and fourth (IV) TMDs, which binds HDV ribonucleoprotein (RNP) via interactions with the large delta antigen. Also indicated are the N-terminus of the PreS1 domain that is critical to infectivity, and the matrix domain in the C-terminus of the PreS1 domain/N-terminus of the PreS2 domain that binds the core antigen of HBV (HBcAg). The myristoyl group attached to the N-terminus of the PreS1 is inserted into the virion membrane (shown as a dark blue rectangle) (1, 4, 5). Also shown is the complete amino acid sequence of the MAL along with the indications of the amino acids that differ at the same positions in the MAL between the HBV genotypes B, C, and D (according to the sequences used in this study). In addition, the HBV IEMs in the MAL, which were examined in this study, are also shown. The numbering used is for the S envelope protein. The topology of the L with the PreS1/PreS2 domains on the outside of the virion represents the infection-competent configuration mediating the binding of the HBV receptor (NTCP) via the N-terminus of the PreS1 domain, while the L topology with the PreS1/PreS2 inside the virion represents the configuration competent for HBV assembly mediating binding of HBcAg to the L envelope protein via the matrix domain (6, 7).

Human hepatitis delta virus (HDV) is a natural sub-viral agent of another significant human pathogen, hepatitis B virus (HBV). HBV provides the envelope proteins (or surface antigen, HBsAg) for HDV. Using HBV envelope proteins, HDV forms its own virions and infects susceptible hepatocytes via the HBV receptor, human sodium taurocholate co-transporting polypeptide (NTCP) (1–7). The HBV-host interactions and anti-HBV immune response facilitate the emergence of the immune escape mutations (IEMs) of HBV. IEMs can mediate the lack of HBV binding by anti-HBsAg antibodies (including HBIg), failure of HBV vaccine to protect against HBV coated with mutated HBsAg, re-infection of grafted liver with HBV, and occult HBV infection. The major antigenic loop (MAL), which is present on all HBV envelope proteins, including the small (S), middle (M), and large (L) envelope proteins, can harbor a wide variety of IEMs throughout its sequence (Fig. 1). The MAL plays an important role in regulating HBV and HDV assembly and infectivity (1, 8–26). While IEMs were previously studied in terms of their regulation of HBV infection, and it was found that IEMs can dramatically impair the secretion of HBsAg and/or HBV virions, and thus can significantly impact the course of HBV infection, the understanding of how IEMs located in the MAL can affect the life cycle of HDV is very limited. Only several IEMs were examined, and it was found that some of them were able to reduce the efficiency of HDV assembly and decrease HDV infectivity (i.e., the amounts of HDV RNA genomes accumulated in infected cells post-infection) (8–14, 23). No studies investigated the influence of the IEMs on the percentage of cells that can be infected with HDV enveloped with IEM-bearing HBsAg, and none of the previous studies was specifically focused on the functional impacts of IEMs and conducted an extensive analysis of the IEM-mediated regulation of the HDV life cycle via examining a considerable number of natural IEMs.

This study examined how 35 different natural IEMs distributed over the entire sequence of the MAL (Table 1; Fig. 1) regulated the assembly and secretion of HDV virions, and influenced their infectivity. In terms of infectivity, we analyzed how IEMs regulated both the number of HDV-infected cells and the number of intracellular HDV RNA genomes produced by HDV infection. We found that all tested IEMs were permissive for HDV assembly and secretion, and only seven IEMs considerably reduced the yield of secreted HDV virions. The effects of IEMs on HDV infectivity were more pronounced. Thirteen out of thirty-five IEMs considerably inhibited HDV infectivity. All these mutations greatly decreased the percentage of HDV-infected cells and the levels of HDV RNA genomes accumulated in the cells post-infection. Overall, our novel findings (i) demonstrated that anti-HBV immune response can down-regulate HDV infection, which potentially could lead to reduction of the size of HDV reservoir in infected livers *in vivo*; and (ii) advanced our understanding of the complex HBV-HDV interactions.

**TABLE 1:** The HBV IEMs selected for the study^*a*^

Mutation	Vaccine-escape mutation	HBIg resistance	Impaired HBsAg secretion	Impaired HBV secretion
Q(101)K				
L(109)I				
I/L(110)M			+	+
S(114)R			−	+
T(116)N	+			
T/A(118)K				+
G(119)E			+	+
**P(120)S**	+			
C(121)Y				
K/R(122)I				
T(123)N				
C(124)R				
T/I(126)N	+	+		
P/T(127)R				
A(128)V				
Q(129)R	+			
G(130)N				
T(131)I	+			
S(132)F				
M(133)T		+	+	&
F/Y(134)V	+			
C(137)R				
C(138)Y			+	+
T(140)S				
**K(141)E**	+	+	+	+
P(142)S	+			
T/S(143)N	+			
D(144)E	+			
**G(145)R**	+	+	+	+
N(146)S			+	+
C(149)R		+	+	+
S(154)L				
A/G(159)C				
F(161)Y				
R(169)P			+	+

^*a*^ All positions are shown using the numbering for the S envelope protein. The existent polymorphism reflecting differences in the amino acid residues between HBV genotypes B, C, and D at the particular positions in the MAL is indicated. Also presented is the known data on the effects of IEMs on secretion of HBsAg and HBV virions. “&” indicates that change 133T actually increases HBV secretion. Also shown are (i) if a particular IEM is the HBV vaccine-escape mutation, and (ii) IEMs conferring resistance to HBIg (hepatitis B immunoglobulin) treatment (HBIg contains anti-HBsAg antibodies and is prepared using plasma from people with high titers of anti-HBsAg antibodies). HBIg is used as anti-HBV immune therapy and is often given to newborns (8–22). Three IEMs (shown in bold), which were previously studied in some aspects for their roles in the HDV assembly and infectivity, serve as internal references. For the IEM S(114)R, the shown effects on the secretion of HBsAg and HBV virions were actually observed for the variant that contained the mutation T(114)R.

## RESULTS

### Influence of IEMs on the assembly of HDV virions

**Fig 2:**
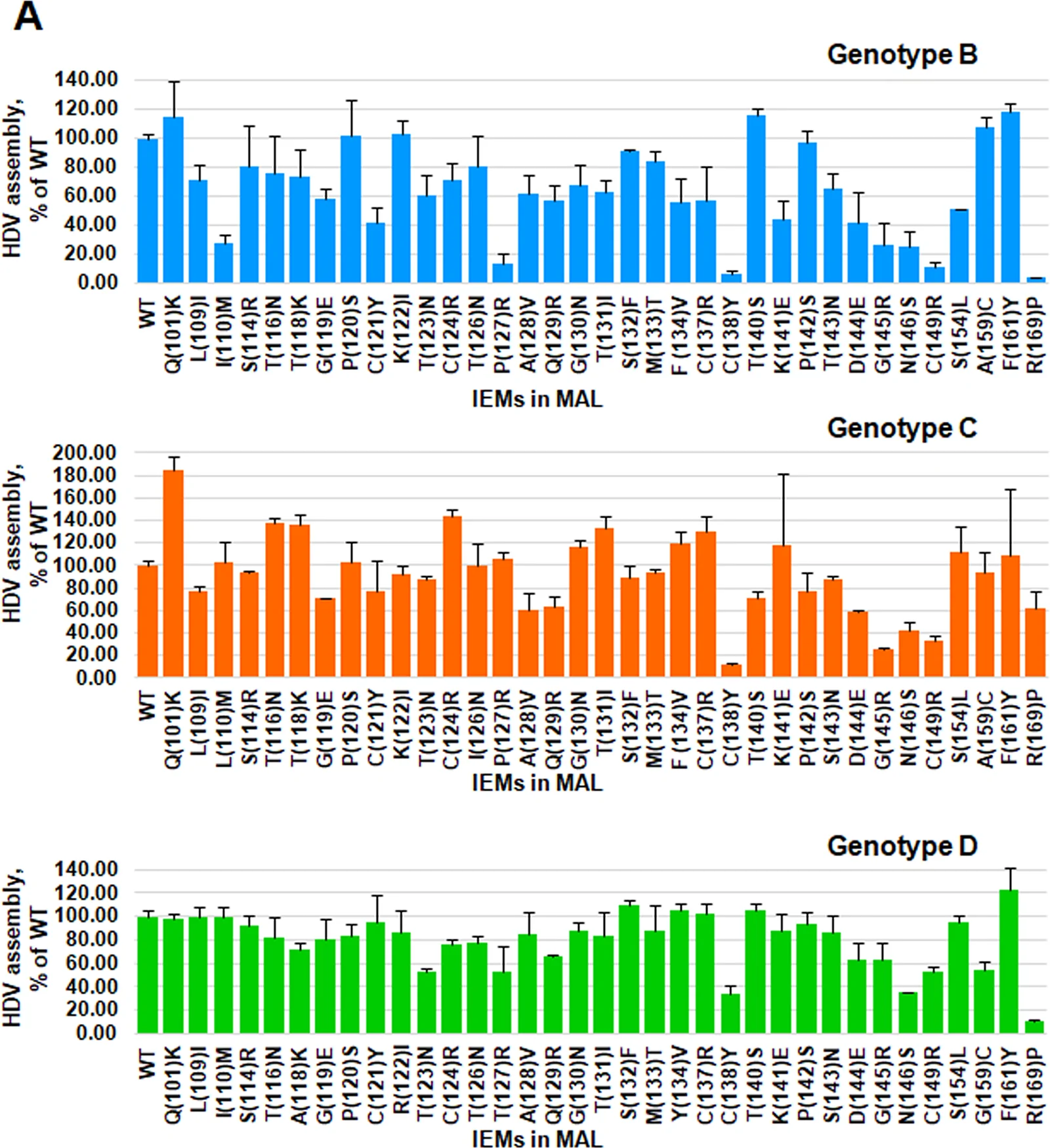

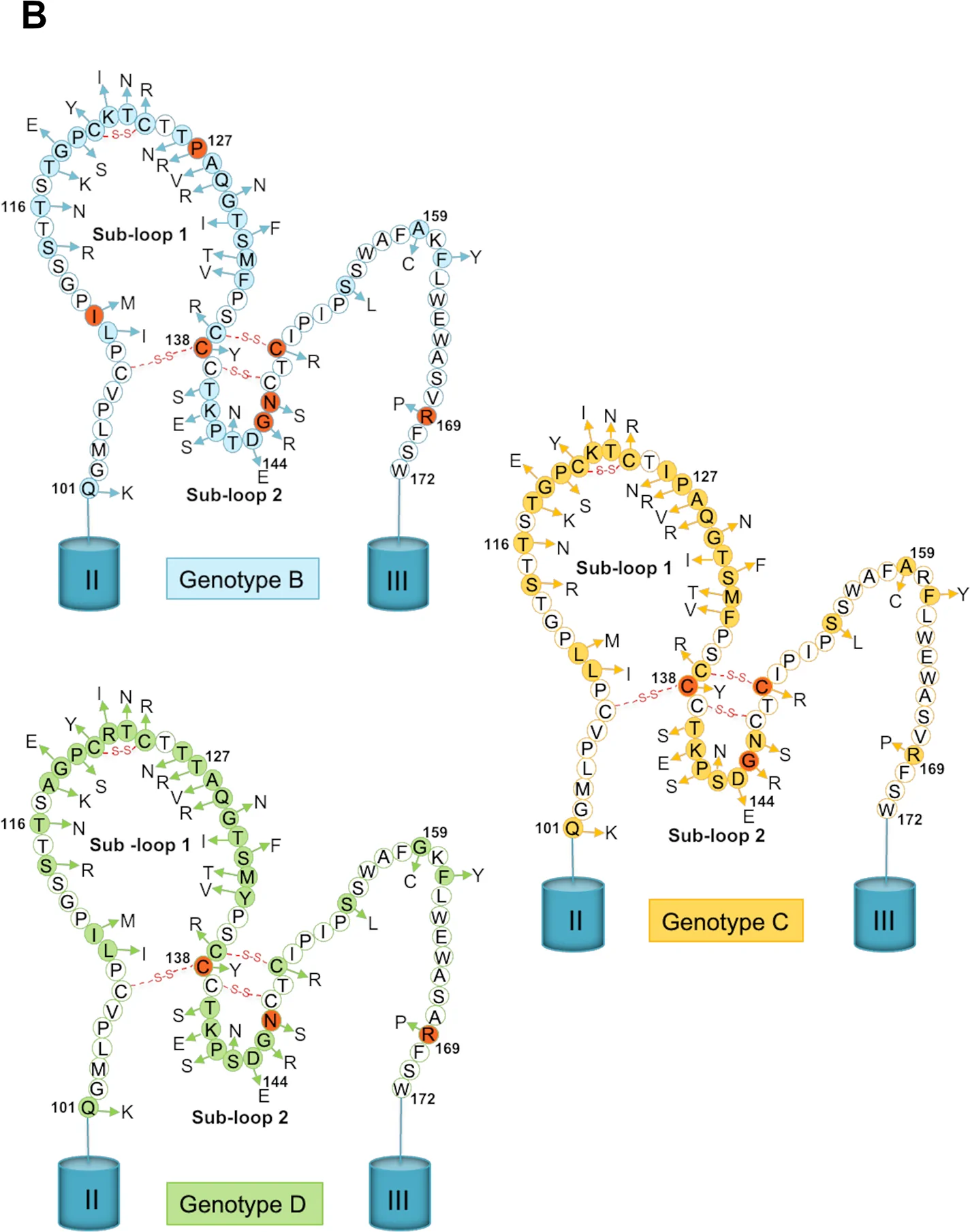
The effects of the immune escape mutations on the assembly of HDV. As detailed in the Materials and Methods, the media were harvested at day 9 post-transfection from Huh7 cells, which were co-transfected with the plasmid pSVLD3 initiating HDV RNA replication along with the HBsAg-expressing vector with or without IEM, and analyzed for the secreted HDV genomic (G) RNA content using RT-qPCR. In panel **A**, the levels of HDV assembly are shown as bars reflecting the percentage relative to the HDV assembly levels achieved using wt HBsAg (without IEMs), which were used as the 100% value. The WT and IEMs are indicated at the bottom of the diagrams. For each set of data on HDV assembly, the corresponding HBV genotype of HBsAg is indicated. In addition, for each of the sets, panel **B** also presents the diagram of the corresponding MAL depicting the IEMs that resulted in significant changes (≥3-fold) in the HDV assembly levels (marked in orange) as compared to the corresponding wt HBsAg. The positions of IEMs are expressed using the numbering of the small (S) envelope protein of HBV. A linear mixed-effects model was used to analyze the difference in assembly levels between WT and IEMs, with IEM as a fixed effect and experiment as a random effect. The HDV assembly levels for all the IEMs, which showed 3-fold or more pronounced decrease in the assembly levels compared to WT, were statistically significant at the 5% significance level (*P* value < 0.05, for each comparison between WT and each IEM) for genotypes B and D, and at the 10% significance level (*P* value < 0.1, for each comparison between WT and each IEM) for genotype C.

We made 35 different natural IEMs in the context of HBsAg, all of which were located in the major antigenic loop, which is present on all three envelope proteins of HBV, large (L), middle (M), and small (S) (Table 1; Fig. 1). To create these mutants, we used three different vectors that drive the expression of all three HBV envelope proteins, L, M, and S (LMS vectors) of the variants that belong to the genotypes B, C, and D of HBV. It needs to be noted that the vector expressing the envelope proteins of genotype C bears the sequence of the natural M-minus HBV mutant and only produces the L and S envelope proteins (i.e., LS vector), which is sufficient to facilitate the assembly and infectivity of HDV and HBV as well (1, 6, 27). The resulting HBsAg-expressing vectors were co-transfected into Huh7 cells along with the pSVLD3 construct initiating HDV genome replication (26–28), and the secreted HDV virions were quantified via assaying HDV RNA genomes in the media at day 9 post-transfection using RT-qPCR (27–29), as detailed in the Materials and Methods. The results reflecting the levels of the secreted HDV virions coated with the HBV envelope proteins (i.e., HBsAg) are presented in Fig. 2.

While the IEMs were located throughout the MAL sequence, none of them was in the HDV-binding site (HDV-BS), which is the stretch of eight amino acid residues located in the small cytosolic loop in the S domain between the third and fourth transmembrane domains (Fig. 1) (1, 4). Therefore, we anticipated that if some of the IEMs could affect the levels of HDV assembly, this would be mediated via an indirect mechanism regulating the efficiency of the formation of HDV virions, and/or interactions of HBsAg with the host factors regulating the efficiency of virion secretion. All the analyzed IEMs were permissive for the HDV assembly and secretion (Fig. 2). The following 28/35 IEMs did not demonstrate a considerable effect on the levels of HDV assembly regardless of the tested HBV genotype: Q(101)K, L(109)I, S(114)R, T(116)N, T/A(118)K, G(119)E, P(120)S, C(121)Y, K/R(122)I, T(123)N, C(124)R, T/I(126)N, A(128)V, Q(129)R, G(130)N, T(131)I, S(132)F, M(133)T, F/Y(134)V, C(137)R, T(140)S, K(141)E, P(142)S, T/S(143)N, D(144)E, S(154)L, A/G(159)C, and F(161)Y. The numbering used is for the S envelope protein. The HDV assembly levels they facilitated were approximately within the range from 42% to 185% as compared to the corresponding wild-type (wt) HBsAg sequence bearing no IEMs (Fig. 2A). The seven of these IEMs (Q(101)K, T(116)N, T(118)K, C(124)R, T(131)I, F(134)V, C(137)R, and F(161)Y) were able to support 120% or higher yields of secreted HDV virions, especially in the context of genotype C of HBV (Fig. 2A). While the levels of HDV assembly mediated by the above-mentioned 28 IEMs varied somewhat between the analyzed IEMs within the context of each of the tested HBV genotypes, and also between the three examined HBV genotypes, the variations reflected less than 3-fold difference from the corresponding wt levels (Fig. 2A). Therefore, the majority of the tested IEMs did not significantly alter the assembly of HDV virions regardless of the HBV genotype, in which context these IEMs were investigated (either B, C, or D).

The remaining 7/35 IEMs reduced HDV assembly levels by about 3-fold or more compared to the levels facilitated by the corresponding wt viral envelope proteins. Only the mutant C(138)Y decreased the levels of secreted HDV virions in all three HBV genotypes examined. The changes G(145)R, C(149)R, and R(169)P decreased HDV assembly in the context of two different HBV genotypes, and the mutants I(110)M, P(127)R, and N(146)S reduced HDV assembly in 1/3 of the tested HBV genotypes (Fig. 2).

In more detail, the change I(110)M reduced HDV assembly by about 3.6-fold, but only for the genotype B of HBV, and not for HBV genotypes C and D. Similarly, the P(127)R decreased HDV assembly by ~7.4-fold for the HBV genotype B without significant effects for the genotypes C and D of HBV. The mutant N(146)S decreased HDV assembly by about 4-fold for the genotype B. The G(145)R reduced the yield of secreted HDV virions by ~3.8–3.9-fold in the context of the HBV genotypes B and C. The C(149)R change led to the reduction of HDV assembly by ~9.6-fold for the HBV genotype B, and by ~3-fold for the genotype C. At the same time, the mutation R(169)P reduced HDV assembly levels by ~27.4-fold in the context of the genotype B, and by ~9.5-fold for the genotype D (Fig. 2). The results for this group of the mutants suggest that IEMs can demonstrate the HBV genotype-specific effects by considerably reducing HDV assembly for some genotypes of HBV, and presenting little or no effect in the context of the other HBV genotypes. In addition, at least in our experimental settings, which considered testing of one HBsAg variant per HBV genotype, it seems that the most sensitive for the effects of IEMs in terms of the efficiency of HDV assembly was the genotype B of HBV (Fig. 2). Finally, the change C(138)Y considerably decreased HDV assembly in the context of all three HBV genotypes, B, C, and D. The C(138)Y reduced the levels of secreted HDV by ~17.3-fold for the genotype B, by ~8.9-fold for the genotype C, and by ~3.0-fold for the genotype D (Fig. 2). The three IEMs, I(110)M, P(127)R, and C(138)Y, considerably decreasing HDV assembly, are located in the first sub-loop of the MAL formed by the S–S bond between Cys107 and Cys138. The next three reducers of HDV assembly are located within the second sub-loop of the MAL, and the remaining mutation R(169)P is situated at the right-hand side of the MAL in the vicinity of the third transmembrane domain (TMD III) (Fig. 2B).

**Fig 3:**
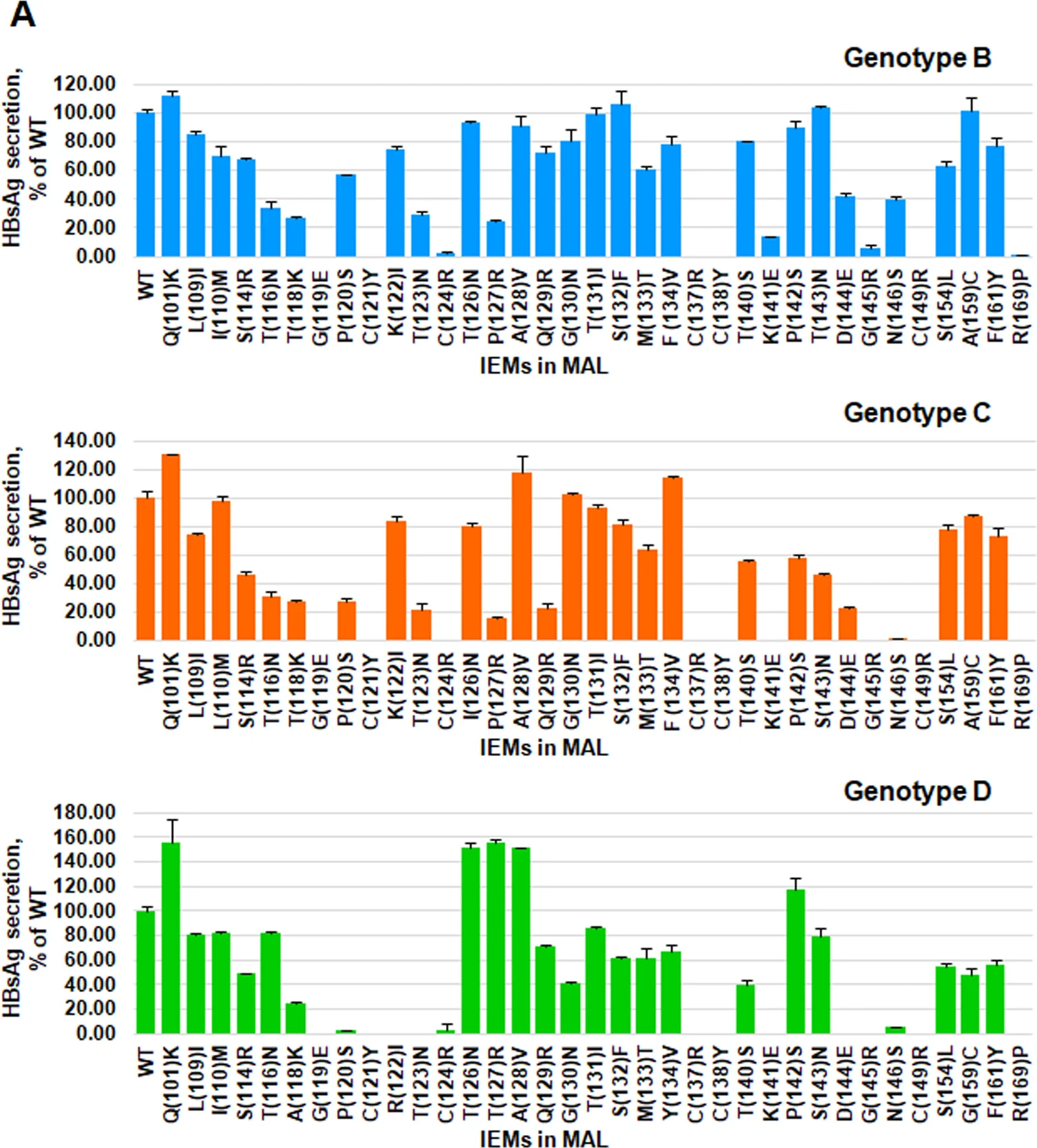

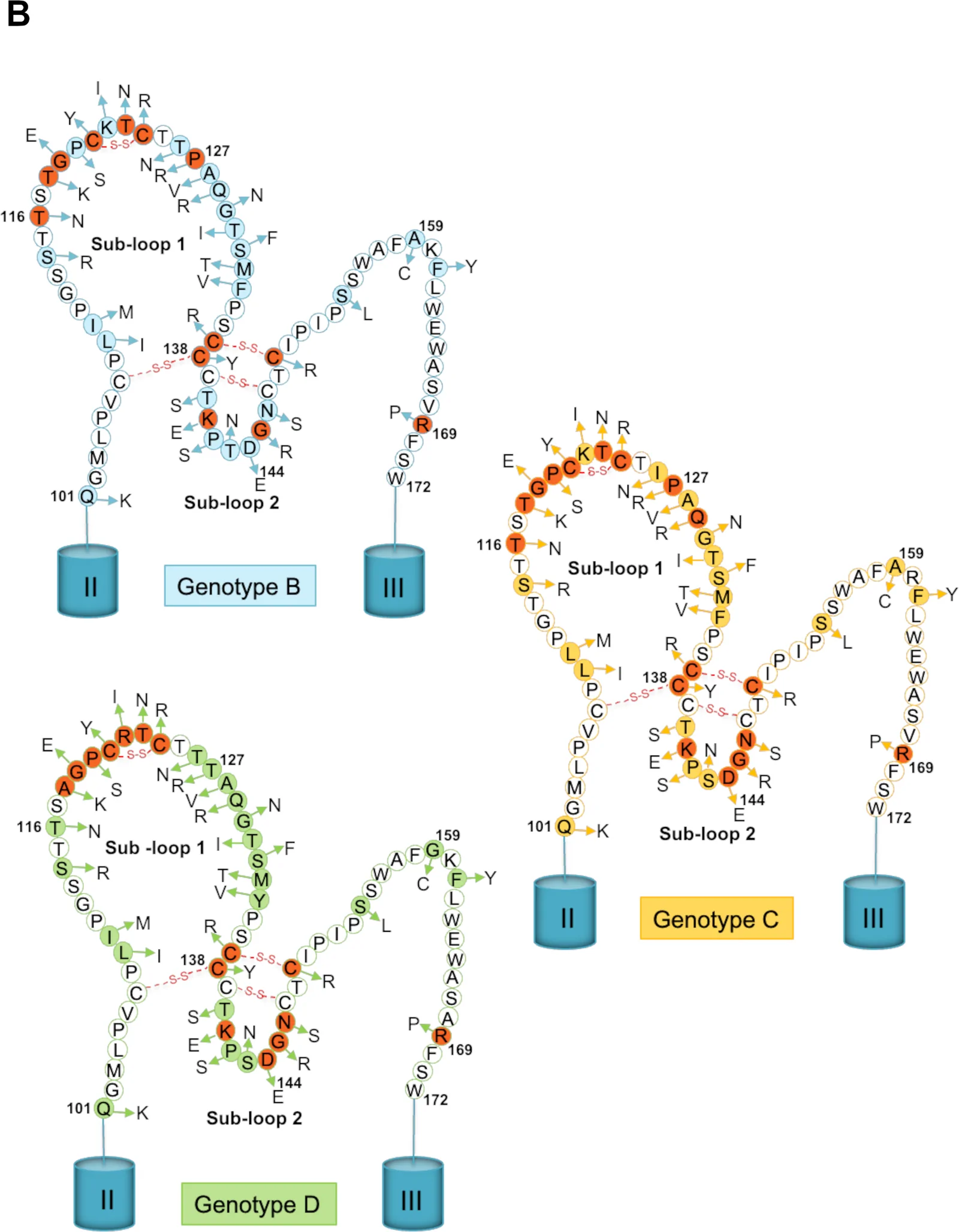
The influence of the immune escape mutations on HBsAg secretion. The media harvested at day 9 after transfection from Huh7 cells, which were co-transfected with the HBsAg-expressing vector with or without IEM along with the plasmid pSVLD3 in order to initiate the assembly of HDV virions, were examined for secreted HBsAg using ELISA (Abbexa). In panel A, the levels of secreted HBsAg are presented as bars reflecting the percentage relative to the HBsAg levels observed using wt HBsAg (without IEMs), which were used as the 100% value. The WT and IEMs are shown at the bottom of each diagram. The analysis was done for the HBV genotypes B, C, and D, as indicated on the diagrams. In panel B, also shown for each tested HBV genotype are the corresponding diagrams of the MAL indicating the IEMs, which resulted in a significant reduction (≥3-fold) of the levels of secreted HBsAg (marked in orange) as compared to wt HBsAg. The positions of IEMs are expressed using the numbering of the small (S) envelope protein of HBV. A linear mixed-effects model was used to analyze the difference in HBsAg secretion levels between WT and IEMs, with IEM as a fixed effect and experiment as a random effect. The HBsAg levels of all the IEMs, which showed 3-fold or more pronounced decrease in HBsAg secretion levels compared to WT, were statistically significant at the 5% significance level (*P* value < 0.05, for each comparison between WT and each IEM) for genotypes B and C, and at the 10% significance level (*P* value < 0.1, for each comparison between WT and each IEM) for genotype D.

In order to get a better understanding of the acquired data regarding the influence of the IEMs on the levels of secreted HDV virions, we conducted the analysis of the levels of secreted HBsAg. We have considered, however, that it has been noted previously that often there was no strict correlation between the levels of HDV assembly and the levels of HBsAg. This is not surprising, because *in vivo* and in the experimental settings of HDV assembly in cell culture, HBsAg is produced in excess, and during egress, most of HBsAg is secreted as empty subviral particles (SVPs), and the minor product of the secretion is HDV virions, for which assembly only a small portion of HBsAg is used. In addition, the HDV assembly will depend on how well a particular variant (or mutant) of HBsAg promotes the formation and secretion of HDV virions (i.e., it rather depends on the functional properties of HBsAg than just on the total amounts of secreted HBsAg) (23, 24, 27, 30, 31). Therefore, it needs to be emphasized that measuring secreted HBsAg during HDV assembly predominantly reflects the levels of empty SVPs not bearing the ribonucleoprotein (RNP) of HDV (consisting of one HDV RNA genome and about 40–200 molecules of the delta antigens [32, 33]), and not necessarily the yield of secreted HDV virions. The secreted HBsAg was measured using HBsAg-specific ELISA (Abbexa). The levels observed for wt HBsAg bearing no IEMs were used as the 100% value for each of the HBV genotypes, and the levels of HBsAg bearing IEMs were expressed as a percentage as compared to the corresponding wt HBsAg. The results of the analysis are presented in Fig. 3. Most IEMs, for which we observed the levels of secreted HBsAg reduced ≥3-fold, were located at the top of sub-loop 1 and in sub-loop 2 of the MAL (Fig. 3B). As shown in Fig. 3, 17/35 of IEMs did not have a significant effect on secreted HBsAg levels. This was the case for the following IEMs in the context of all three tested HBV genotypes: Q(101)K, L(109)I, I(110)M, S(114)R, T/I(126)N, A(128)V, G(130)N, T(131)I, S(132)F, M(133)T, F/Y(134)V, T(140)S, P(142)S, S/T(143)N, S(154)L, A/G(159)C, and F(161)Y. All the observed differences were less than 3-fold compared to the wt HBsAg. For these IEMs, the levels of secreted HBsAg and the levels of HDV assembly both were considerable and did not differ significantly (≤3-fold difference) from the corresponding wt levels. The only exception was IEM I(110)M, which caused the decrease of HDV assembly by about 3.6-fold only for genotype B of HBV (Fig. 2 and 3).

While HBsAg levels were reduced by about 3–4-fold (for at least 1/3 tested HBV genotypes), the HDV assembly remained unaffected for the following IEMs: T(116)N, T(118)K, and Q(129)R. For the next group of IEMs, the measured HBsAg levels were either dramatically reduced or were undetectable for at least 1/3 HBV genotypes, but HDV assembly was not changed considerably. These IEMs were G(119)E, P(120)S, C(121)Y, K(122)I, T(123)N, C(124)R, C(137)R, K(141)E, and D(144)E (for this IEM, however, the levels of secreted HBsAg and HDV assembly correlated, but only in the case of genotype B, and not for genotypes C and D). In addition, previous reports also showed reduced yield of secreted HBsAg for T(118)K, G(119)E, and K(141)E (10–12, 23). We considered that the positions C121, C124, C137, and K141 were shown to be important for the maintenance of the antigenicity of the MAL (23). Therefore, for these positions, our measured HBsAg levels, at least in a large part, could be explained by the altered recognition of the mutation-bearing HBsAg variants by the anti-HBsAg antibodies. As for the other positions, significantly reduced measured HBsAg levels most likely reflect the low yield of secreted HBsAg, because for those positions in the MAL, it was not reported that they were important for the antigenicity (23). It is, however, clear that for these latter two groups of IEMs, there was enough HBsAg produced and secreted to ensure considerable levels of HDV assembly (Fig. 2 and 3).

The mutant R(169)P was previously shown to be severely defective in HBsAg secretion, while no major defects were found in terms of the intracellular accumulation of R(169)P-bearing HBsAg (10). We also measured extremely low or undetectable secreted HBsAg for R(169)P (Fig. 3), which is consistent with greatly reduced HDV assembly for genotypes B and D (Fig. 2). At the same time, for genotype C, the finding that R(169)P-bearing HBsAg was undetectable by ELISA and the HDV assembly levels were at about 62% as compared to the wt HBsAg-enveloped HDV indicates that even in this case, there was enough HBsAg to facilitate a considerable yield of secreted HDV virions (Fig. 2). In this case, the alteration of the HBsAg antigenicity is unlikely because the position R169 was reported as the one not critical for maintaining the antigenicity of the MAL (23). Next, we examined the changes C(149)R and C(138)Y. Interestingly, an earlier report did not demonstrate significant defects in the intracellular accumulation of HBsAg bearing these mutations (12). The change C(149)R, however, was shown to reduce the secretion of HBsAg to the levels of approximately from several percent to about 30% as compared to wt HBsAg (depending on the assay used) (12), which at least in part could reflect the loss of HBsAg antigenicity that was previously shown for the mutations C(149)S and C(149)A in this position (23, 34). We observed undetectable HBsAg via ELISA for the change C(149)R for all tested HBV genotypes (Fig. 3), which apparently was somewhat consistent with greatly reduced HDV assembly for genotype B and about 3-fold decreased HDV assembly for genotype C of HBV (Fig. 2). However, the decreased less than 2-fold HDV assembly for genotype D (Fig. 2) could suggest that in this case, there was enough of functional HBsAg produced to achieve the yield of secreted HDV virions of about 54%. At the same time, in this case, the ELISA results could likely at least in a large part be explained by reduced recognition by the anti-HBsAg antibodies of the HBsAg bearing the C(149)R mutation in the context of the genotype D (Fig. 2). The follow-up studies could consider investigating if in the context of the genotypes B and C, the change C(149)R led to reduced HBsAg levels, and/or perhaps it also changed the properties of the envelope proteins in terms of the formation of HDV virions. The IEM C(138)Y considerably reduced HDV assembly in the context of all three HBV genotypes, but had a more pronounced effect for the genotypes B and C (Fig. 2), and for this IEM, we found secreted HBsAg to be undetectable by ELISA (Fig. 3). These findings are similar to the previously reported dramatic reduction of the HBsAg secretion caused by this change of the Cys to Tyr at position 138 (12). However, since C138 was also previously reported as a critical residue for the MAL antigenicity (23), a good possibility here is that the loss of the antigenicity at least partially explains the ELISA results, and that alteration of HBsAg properties at least in part led to reduced HDV assembly. Therefore, to better understand the observations regarding IEM C(138)Y, the follow-up studies could address in more detail the properties of HBsAg variants bearing this mutation. The change G(145)R was previously reported as the mutation that was able to decrease intracellular levels of HBsAg and also to considerably reduce HBsAg secretion to the levels of about 20%–25% (10, 12), while we found HBsAg to be undetectable for the genotypes C and D, and only at levels close to 6% as compared to wt HBsAg for genotype B (Fig. 3). The G(145)R was also shown to reduce the antigenicity of the MAL to the level of 20% compared to the wt HBsAg (23). Thus, our results on HBsAg detection could be in part due to reduced recognition by the anti-HBsAg antibodies. Interestingly, this IEM was reported to be associated with the inability to detect HBsAg in patients (35). We observed for this IEM, the decreased HDV assembly for the genotypes B and C, while no major influence was observed for genotype D (Fig. 2). Considering the observed by us (Fig. 3) along with the previously reported substantial reduction of secreted HBsAg shown by different methods (10, 12, 23), the reduction by about 4-fold of the yield of secreted HDV virions for the IEM G(145)R (Fig. 2) is likely largely due to diminished HBsAg levels (Fig. 3A).

For the mutant N(146)S, no considerable reduction of the levels of intracellular and secreted HBsAg was reported before (12). Observed by us the reduction of HDV assembly by ~4-fold only for genotype B (Fig. 2) did not exactly correlate with the measured HBsAg levels by ELISA of ~40% (Fig. 3). Therefore, in this case, it is likely that the alteration of HBsAg functioning and not HBsAg yield *per se* could be the major contributor to the decreased HDV assembly levels. At the same time, the dramatically decreased HBsAg levels to ~1%–5% for the HBV genotypes C and D (Fig. 3A) did not result in significant changes of the HDV assembly levels, which were ≤3-fold, and thus were not considered as substantial (Fig. 2A). The mutation I(110)M did not cause any considerable decrease in the levels of secreted HBsAg (Fig. 3A). For this IEM, no dramatic decrease of intracellular HBsAg and no great reduction of HBsAg secretion was found previously as well (10). Thus, the reduction of HDV assembly by about 4-fold observed only for genotype B (Fig. 2) is likely the result of altered HBsAg properties. The change P(127)R resulted in reduced HBsAg secretion by ~4- to 6-fold for genotypes B and C (Fig. 3), but decrease of HDV assembly levels was only observed for genotype B by ~7-fold (Fig. 2). Such decrease is more pronounced than the decrease in HBsAg levels, and it is likely that the alteration of the functioning of HBsAg was a considerable contributor diminishing the yield of secreted HDV. Thus, the reduction of HDV assembly observed for the three above-mentioned IEMs is likely mainly due to the IEM-induced changes of the functional properties of the viral envelope proteins.

Overall, for some above-mentioned IEMs, the main contributing factor to reduced HDV assembly was decreased levels of secreted HBsAg, while for other cases it was most likely alteration of the properties of the HBV envelope proteins. There were also some IEMs for which both those factors could be the reasons for decreased levels of secreted HDV virions. In addition, as described above, we found that the same IEM could affect the HBsAg levels to very different extents in the context of different HBV genotypes. Several examples of that are (i) T(116)N reduced HBsAg levels by about 3-fold only for genotypes B and C, but not for genotype D; (ii) Q(129)R reduced HBsAg secretion by ~4-fold only for genotype C, and not for genotypes B and D; (iii) P(120)S caused substantial reduction of HBsAg levels only for genotypes C and D, but not for B; and (iv) N(146)S led to markedly reduced HBsAg levels only for genotypes C and D, but not for genotype B (Fig. 3). These findings represent some of the HBV genotype-specific effects of the tested IEMs.

**Fig 4:**
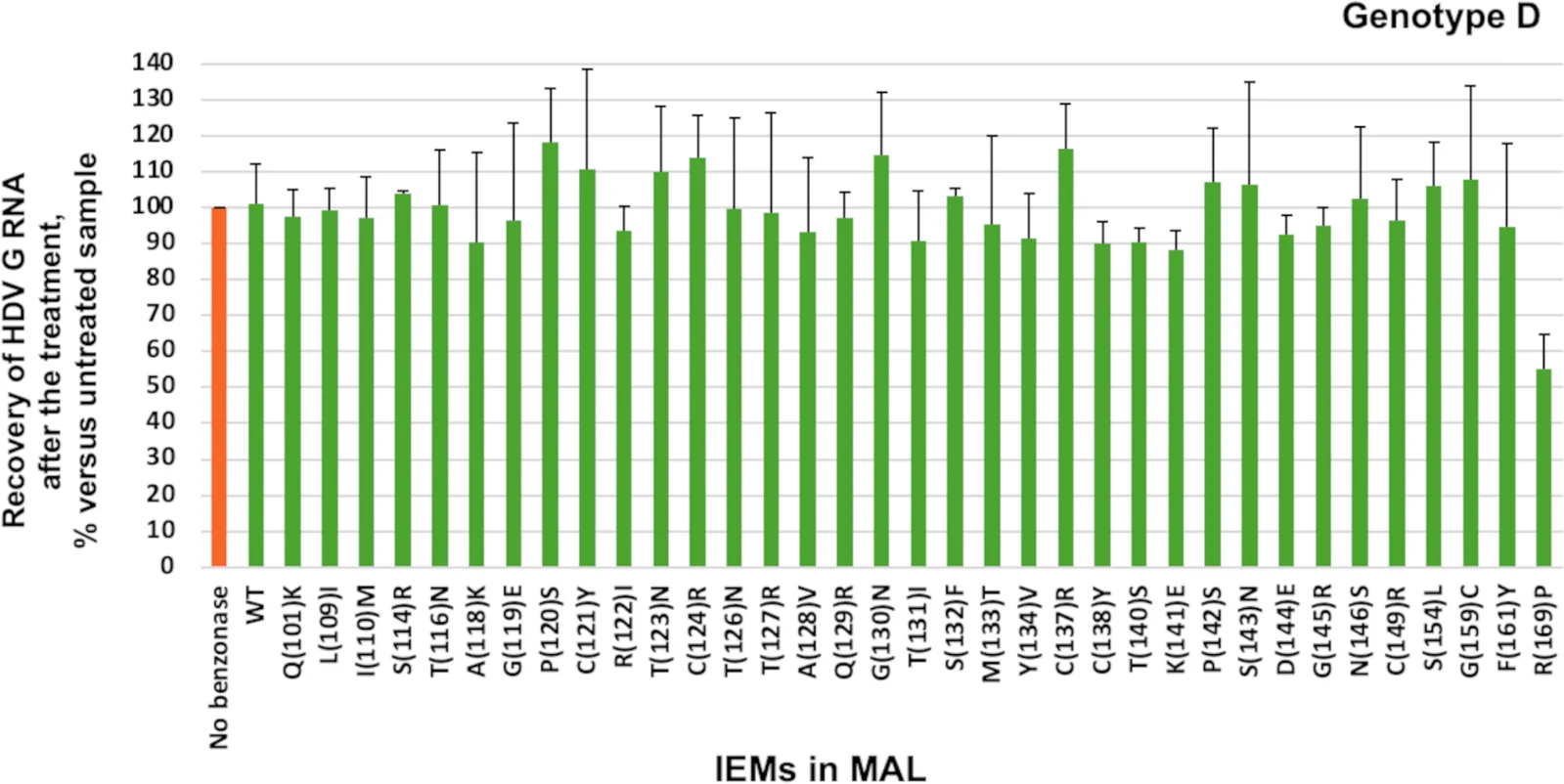
Benzonase-protection analysis of secreted HDV RNA genomes. The treatment with the benzonase nuclease of the media samples bearing the HDV G RNA secreted during the assembly of HDV virions was done as detailed in the Materials and Methods. We analyzed the HDV virion preparations for the HBsAg of the HBV genotype D. The percentage of recovery of the HDV G RNA after the benzonase treatment is displayed as the percentage relative to the corresponding control reactions (for each sample) that were conducted in the absence of the benzonase nuclease. The recovered levels of the HDV G RNA from those control reactions were used as the 100% value. The IEMs are indicated at the bottom of the figure. A linear mixed-effects model was used to analyze the difference in the percentage of recovered HDV G RNA levels after the benzonase treatment between WT and IEMs, with IEM as a fixed effect and experiment as a random effect. Only mutation R(169)P showed a statistically significant reduction in the percentage of recovered HDV G RNA levels compared to WT, with a *P* value < 0.0001. This IEM was excluded from further statistical analysis. Next, a one-sample *t*-test was used to analyze the difference between benzonase-treated and benzonase-untreated samples (the latter were represented as 100% value). At a 5% significance level, there is enough evidence to claim that there is no statistically significant difference between benzonase-treated and benzonase-untreated samples (represented as 100% value) with *P* value = 0.89 and 95% confidence interval [96.72; 102.86].

In addition, we also examined whether the secreted HDV RNA is resistant to treatment with the benzonase nuclease, which was expected to degrade all the forms of unprotected DNA and RNA species present in the sample. We analyzed the HDV virion preparations for the HBsAg of the HBV genotype D. Previously, we used the benzonase nuclease treatment to analyze the integrity of the circulating in the blood HDV virions in samples collected from the woodchucks that were chronically infected with the closely related to HBV — woodchuck hepatitis virus (WHV), and then they were super-infected with WHV-enveloped HDV, and in the current study, we employed the protocol of the benzonase treatment used for the woodchuck serum samples (36). The results of this analysis are summarized in Fig. 4. As can be seen, in all cases, between 88.38% and 118.26% of HDV G RNA that was in the virion’s preparations (i.e., secreted into the media) were recovered after the benzonase treatment (Fig. 4), while purified from the virions, HDV G RNA was predominantly destroyed by the same treatment (not shown). Such protection of RNA from the nuclease is expected for the HDV G RNA being inside the virions, whose integrity is not compromised. There was only a single exception. For the IEM R(169)P, the average percentage for the recovery of the HDV G RNA protected from the benzonase treatment was 55.11% (Fig. 4). One explanation here is that this finding is consistent with the altered functioning of HBsAg mediated by IEM R(169)P resulting in structural alterations of the virion’s envelope that produced a considerable fraction of defective virions, which did not ensure the proper protection of their internal cargo (i.e., HDV RNP).

### Regulation of HDV infectivity by the IEMs present in HBV envelope proteins

The MAL (Fig. 1) can participate in attachment/entry by interacting with cell surface glycosaminoglycans as part of the initial attachment of the virion to the outer membrane of the susceptible cell and can play a certain role during the intracellular post-entry trafficking of the virions. It also possibly could be involved in the intracellular disassembly process (uncoating or loss of the virion’s envelope) (23, 27), which will affect the overall infectivity of HDV virions. The effects of IEMs on HDV infectivity were examined using HBsAg of the genotype D of HBV. We therefore prepared, as previously described (27, 28), the concentrated stocks of the HDV virions with and without IEMs in HBsAg coating the viral particles, and analyzed the infectivity of these HDV virions using *in vitro* infection of HepG2-NTCP cells, bearing the HBV receptor, sodium taurocholate co-transporting polypeptide (NTCP), and thus susceptible to the HBV and HDV infections, and supporting the complete life cycles for these two viruses (2, 34, 37). In these experimental settings, the tested IEMs were present on all three HBV envelope proteins, the L, M, and S. As the negative control, we used HDV assembled with the S envelope protein only, which was not infectious (27). The cells were analyzed at day 8 post-infection.

**Fig 5:**
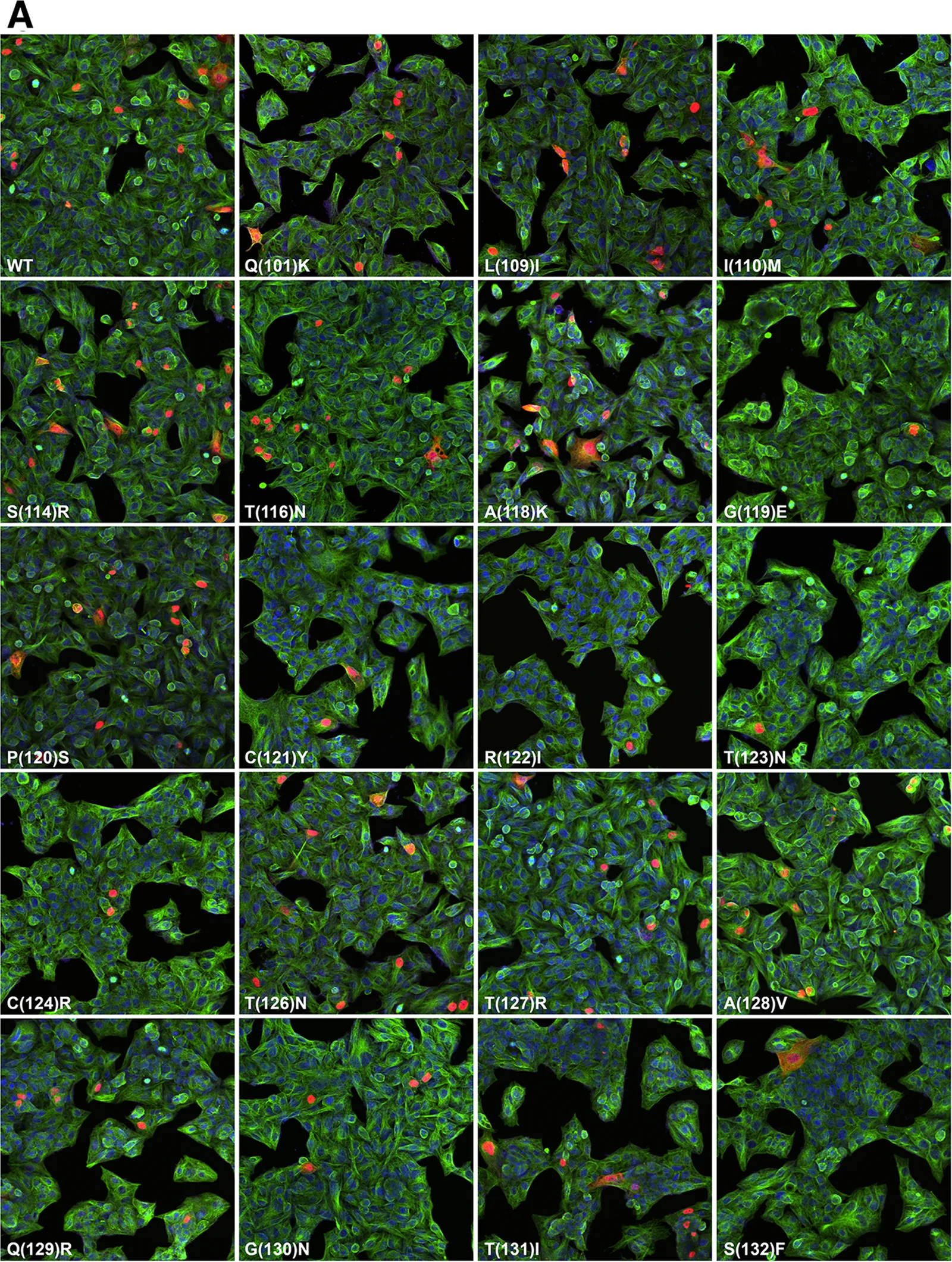

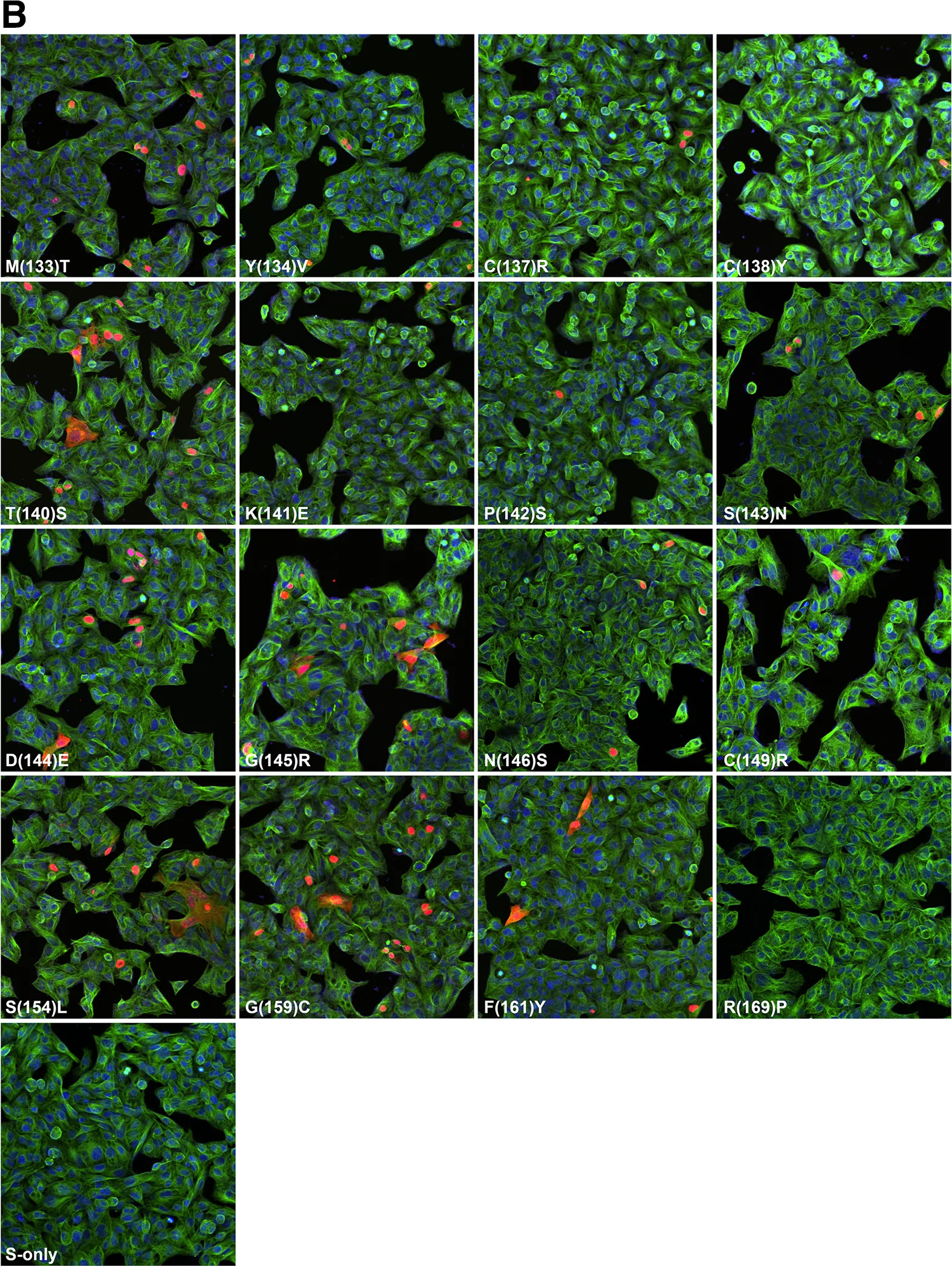
HepG2-NTCP cells infected with HDV virions coated with HBV envelope proteins bearing IEMs. The figure demonstrates the images of HepG2-NTCP cells infected with HDV virions enveloped with different variants of HBsAg of HBV genotype D, which bear or do not bear IEMs. Each image indicates the type of HBsAg that contained or did not contain IEM, which coated the HDV virions in the corresponding experiment. The wt reflects HBsAg not bearing IEMs (the control). The cells were analyzed at day 8 post-infection with HDV using immunofluorescence as detailed in the Materials and Methods. The red staining is for the delta antigens, the green staining is for the cellular alpha-tubulin, and the blue staining is for nuclear DNA (DAPI). The different patterns of intracellular distribution of the delta antigens generated by HDV infection are analyzed in Fig. 6 and 7 and discussed in the text. The fraction of the delta antigen-positive (i.e., HDV-infected cells) was used to calculate the relative percentage of the infected cells (see text). Panel **A** shows the results for the wt, and the following IEMs: Q(101)K, L(109)I, I(110)M, S(114)R, T(116)N, A(118)K, G(119)E, P(120)S, C(121)Y, R(122)I, T(123)N, C(124)R, T(126)N, T(127)R, A(128)V, Q(129)R, G(130)N, T(131)I, and S(132)F. Panel **B** displays the results for the IEMs M(133)T, Y(134)V, C(137)R, C(138)Y, T(140)S, K(141)E, P(142)S, S(143)N, D(144)E, G(145)R, N(146)S, C(149)R, S(154)L, G(159)C, F(161)Y, R(169)P, and for the control that used non-infectious virions coated with the wt S envelope protein only (i.e., S-only) (27).

**Fig 6:**
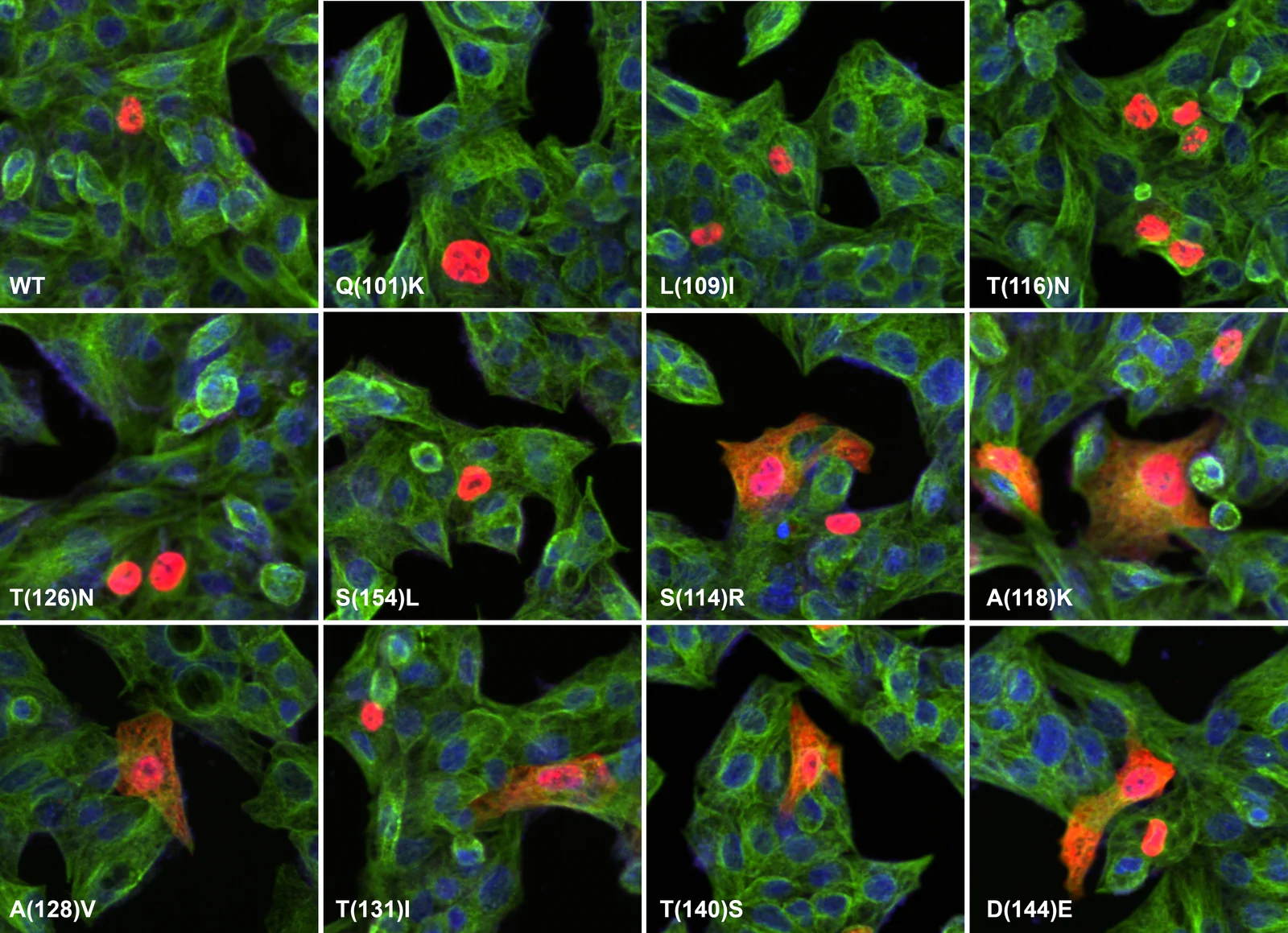
Patterns of the intracellular distribution of the delta antigens in the HDV-infected HepG2-NTCP cells. The figure displays two types of patterns of the intracellular distribution of the delta antigens in the HDV-infected HepG2-NTCP cells — major and minor. The images are shown using a higher magnification as compared to the images in Fig. 5. The major pattern reflects the delta antigens localized in the nucleoplasm with nucleoli exclusion. This pattern is shown on the representative images for the wt and the following IEMs: Q(101)K, L(109)I, T(116)N, T(126)N, S(154)L, S(114)R, A(118)K, T(131)I, and D(144)E. The minor pattern reflected by the cytoplasmic staining for delta antigens along with the staining of the nucleoplasm with nucleoli exclusion is shown for the S(114)R, A(118)K, A(128)V, T(131)I, T(140)S, and D(144)E. The images for the S(114)R, A(118)K, T(131)I, and D(144)E display both the above-mentioned patterns.

**Fig 7:**
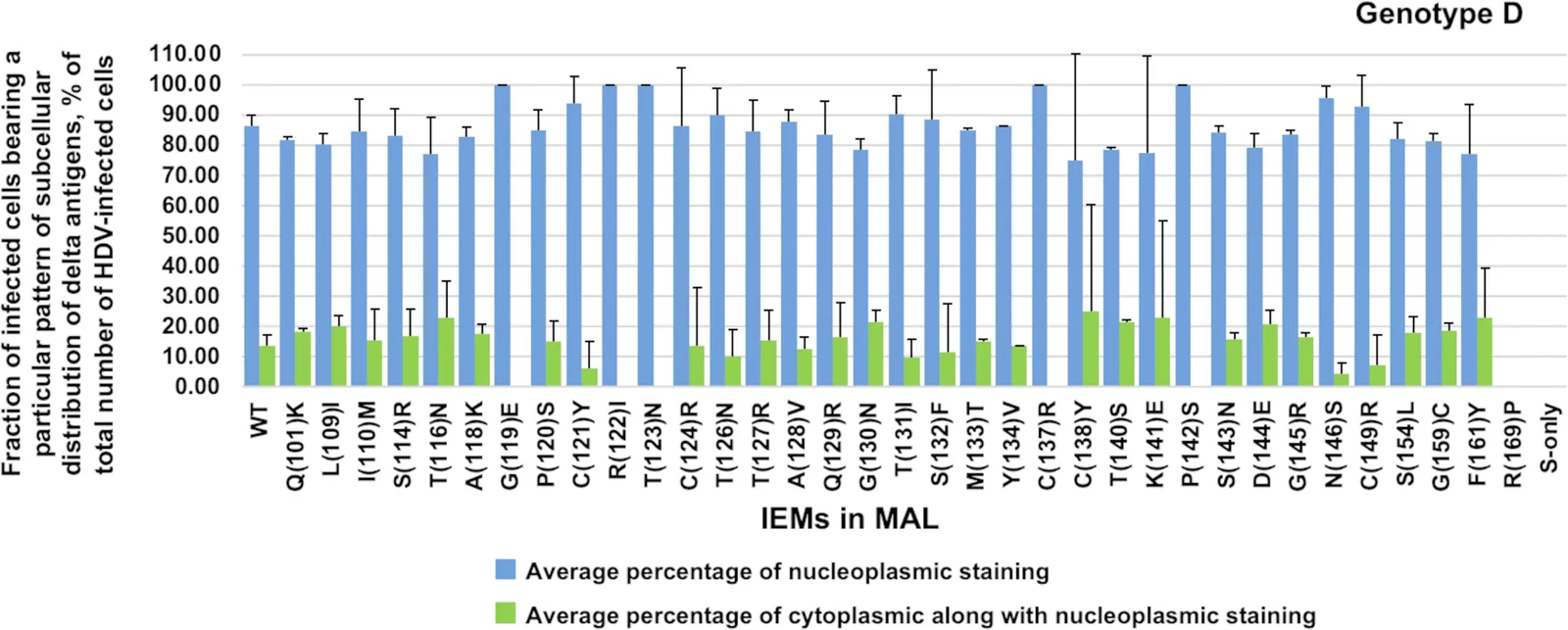
Fractions of HDV-infected HepG2-NTCP cells displaying one or the other of the two common patterns of the intracellular distribution of delta antigens. As mentioned in the legend to Fig. 6, the major common pattern of the intracellular localization of the delta antigens in the infected cells is the diffuse nucleoplasmic staining with nucleoli exclusion, while the minor common pattern is the staining of the cytoplasm along with the nucleoplasmic staining. Shown is the fraction of the HDV-infected cells exhibiting either of the above-described major or minor patterns of the intracellular distribution of the delta antigens (for each of the tested IEMs) as the percentage of the total number of HDV-infected cells. The percentage for the nucleoplasmic staining with nucleoli exclusion is shown as blue bars, while the percentage for the cytoplasmic along with the nucleoplasmic staining is displayed as green bars. The total number of infected cells in each case that represented all HDV-infected cells positive for delta antigens regardless of the pattern of the intracellular distribution of the delta antigens was used as a 100% value. For the IEMs, G(119)E, R(122)I, T(123)N, C(137)R, and P(142)S, the minor pattern of the intracellular distribution of the delta antigens in infected cells was not observed. The ratios for the nuclear to (cytoplasmic plus nuclear) stainings for the delta antigens were calculated per replicate with a small pseudocount (+0.5) added to avoid zero counts. After log transformation, a one-sample *t*-test against 0 (corresponding to equal nuclear and [cytoplasmic plus nuclear] distribution of the delta antigens in each sample reflecting the experiment for one IEM) showed significant nuclear distribution enrichment (mean log ratio = 0.91, *P* value < 0.0001), corresponding to an approximately 8.1-fold higher numbers of nuclear localization (on average).

We have examined two aspects of the HDV virions’ infectivity. Using immunofluorescence and staining for the delta antigens, we analyzed the fraction of HDV-infected cells (27, 28). The infection experiments performed for the analysis of the percentage of HDV-infected HepG2-NTCP cells were done using the multiplicity of infection (MOI) of 100 HDV genome equivalents per cell (100 HDV GE/cell). The MOI is the number of HDV genome equivalents (GE) per cell in the inoculum used for the infection. The images of the HDV-infected HepG2-NTCP cells for each of the IEMs are presented in Fig. 5. The HDV infection usually produces two patterns of the intracellular distribution of the delta antigens in the infected cells, as presented for the selected infections in Fig. 6. The most frequent pattern of the subcellular localization of the infection-derived delta antigens was diffused staining throughout the nucleoplasm with the exclusion of nucleoli (Fig. 6). Its representative examples are shown for WT, and for Q(101)K, L(109)I, T(116)N, T(126)N, S(154)L, S(114)R, A(118)K, T(131)I, and D(144)E. On much less frequent occasions, we also observed another pattern, when delta antigens were located both in the nucleoplasm and also in the cytoplasm (Fig. 6). The representative examples of this pattern are shown for S(114)R, A(118)K, A(128)V, T(131)I, T(140)S, and D(144)E. The images for the IEMs, S(114)R, A(118)K, T(131)I, and D(144)E, demonstrate both above-mentioned patterns. We also calculated the relative percentages of the HDV-infected cells that displayed either of the above-described patterns of the intracellular localization of the delta antigens for the infections conducted with control HDV virions coated with wt HBsAg and for the infections that used HDV virions enveloped with IEM-bearing HBsAg. Clearly, the majority of the infected cells in each infection displayed the staining for the delta antigens in the nucleoplasm with nucleoli exclusion (Fig. 7) that was described above as the major pattern for intracellular distribution of the delta antigens. For the IEMs, G(119)E, R(122)I, T(123)N, C(137)R, and P(142)S, only the above-mentioned major pattern was found, and the minor pattern was not observed (Fig. 7). Both these patterns were previously reported by us for HDV infection of primary human hepatocytes (27, 31). To conduct further comparative analysis, we expressed the data on the fraction of the HDV-infected HepG2-NTCP cells as the percentage relative to the fraction of the infected HepG2-NTCP cells, which was observed for the infection conducted using HDV virions coated with wt HBsAg not containing IEMs, and which was used as the 100% value. Next, we also isolated total RNA from infected HepG2-NTCP cells using TRI-reagent, and using the extracted RNA we measured the levels of intracellular HDV genomic (G) RNA generated by the infection via RT-qPCR (27, 38). For the subsequent comparative analysis, the accumulation levels of the intracellular infection-generated HDV G RNA were expressed as the percentage relative to the levels of HDV G RNA observed for the HDV virions coated with wt HBsAg not bearing IEMs, which were used as the 100% value. All infections for the analysis of the levels of HDV G RNA in the infected HepG2-NTCP cells were done using the same MOI of 70 (HDV GE/cell).

**Fig 8:**
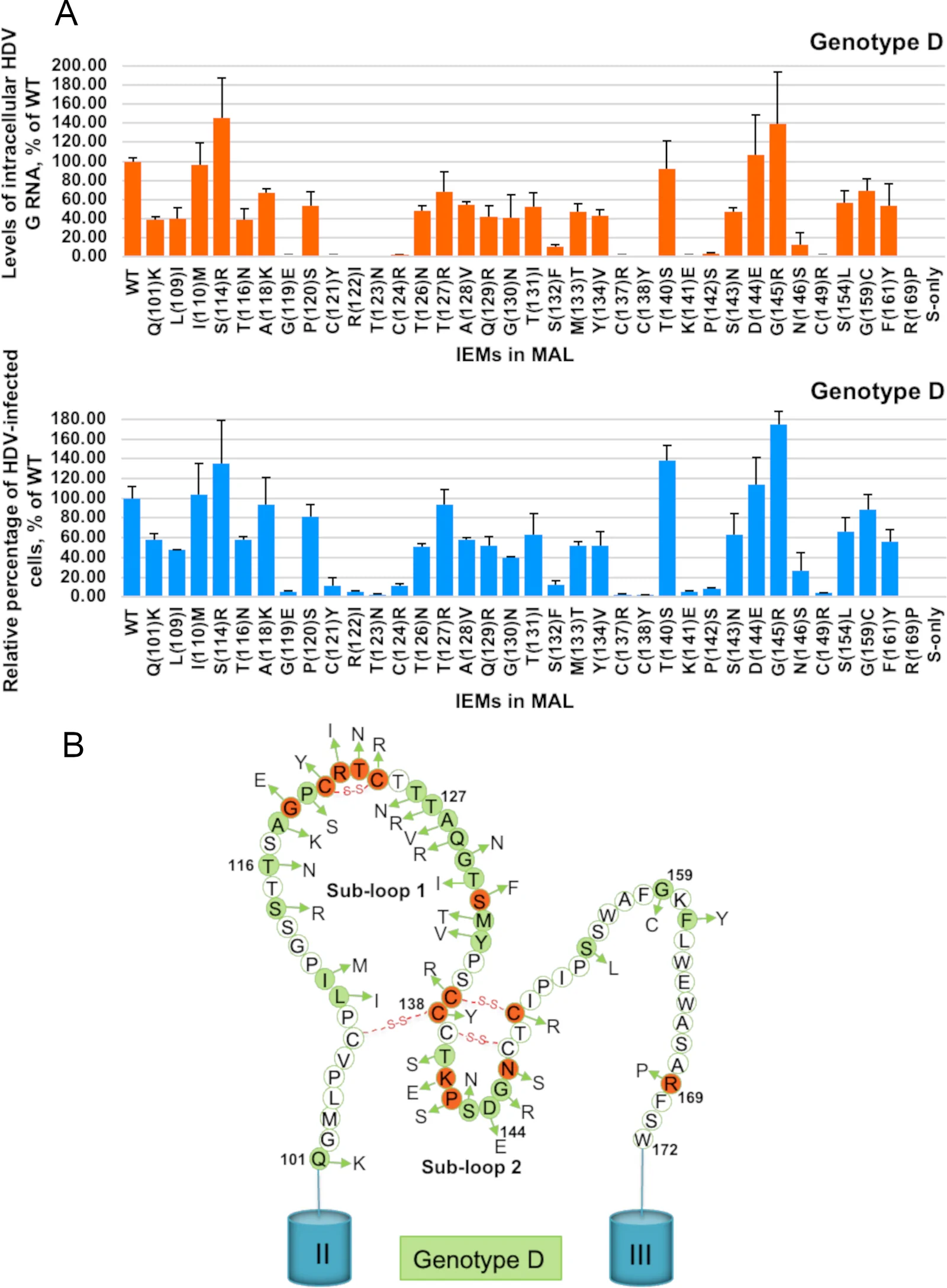
Analysis of the infectivity of HDV virions coated with HBV envelope proteins bearing IEMs. Two aspects of the infectivity of HDV virions coated with HBsAg variants of the HBV genotype D that bear or do not bear IEMs were analyzed, and the results are shown in panel **A**. First, we assayed (using RT-qPCR) the levels of the intracellular accumulation of the infection-generated HDV G RNA in infected HepG2-NTCP cells, which for the purposes of the comparative analysis are expressed as the percentage relatively to the levels of HDV G RNA observed in the cells, which were infected with HDV virions coated with wt HBsAg (not bearing IEMs) (shown on the top of panel **A**). The levels of the HDV G RNA in infected cells found for the HDV virions enveloped with wt HBsAg were used as the 100% value. Second, we used immunofluorescence to calculate the fraction of the HDV-infected HepG2-NTCP cells, and then, for the purposes of the comparative analysis, expressed the data relatively to the fraction of the infected cells observed for the infection, which was conducted using HDV virions enveloped with wt HBsAg (not bearing IEMs). Thus, the figure displays the relative percentage of the HDV-infected cells (on the bottom of panel **A**). The percentage of the HDV-infected cells found for the HDV virions coated with wt HBsAg was used as the 100% value. The WT and IEMs are indicated at the bottom of the diagrams. In panel **B**, the IEMs that significantly reduced both of the above-mentioned aspects of the infectivity are marked in orange on the corresponding MAL diagram. A linear mixed-effects model was used to analyze the difference in the levels of intracellular HDV G RNA between WT and IEMs, with IEM as a fixed effect and experiment as a random effect. The levels of HDV G RNA for all the IEMs, which showed 3-fold or more pronounced decrease in the levels of intracellular HDV G RNA as compared to WT, were statistically significant at the 5% significance level (*P* value < 0.05 for each comparison between WT and each IEM). In addition, a linear mixed-effects model was used to analyze the difference in the relative percentage of HDV-infected cells between WT and IEMs, with IEM as a fixed effect and experiment as a random effect. The relative percentage of the infected cells for all the IEMs, which showed 3-fold or greater decrease in the relative percentage of HDV-infected cells as compared to WT, was statistically significant at the 1% significance level (*P* value < 0.01 for each comparison between WT and each IEM).

The results of the analysis of the relative levels of HDV G RNA and the relative percentage of the HDV-infected cells are summarized in Fig. 8. The 22 out of 35 IEMs had no significant effect on the infectivity of HDV virions. These mutations were Q(101)K, L(109)I, I(110)M, S(114)R, T(116)N, A(118)K, P(120)S, T(126)N, T(127)R, A(128)V, Q(129)R, G(130)N, T(131)I, M(133)T, Y(134)V, T(140)S, S(143)N, D(144)E, G(145)R, S(154)L, G(159)C, and F(161)Y. The changes in the values of the levels of HDV G RNA and/or the relative percentage of the infected cells observed for them reflected less than 3-fold differences as compared to the corresponding values supported by wt HBsAg. Interestingly, for S(114)R, D(144)E, and G(145)R mutants, both values of the levels of HDV G RNA and the relative percentage of the infected cells were somewhat higher than those found for wt HBsAg. In addition, the change T(140)S resulted in the relative percentage of the infected cells being higher than that observed for the wt HBsAg. While these increases in the levels of HDV G RNA and/or relative percentage of the infected cells for these IEMs were apparent, they still differed less than 2-fold from the corresponding values observed for the wt HBsAg. For the rest of these IEMs, the levels of HDV G RNA were in the range from 38% to 97%, and the relative percentage of the infected cells was in the range of 39% to 104% as compared to those found for the wt HBsAg (Fig. 8A).

The remaining 13/35 IEMs notably affected HDV infectivity. Only one mutant R(169)P had no measurable values for the levels of HDV G RNA and for the relative percentage of the infected cells (Fig. 8), and not a single HDV-infected cell was found in this case (Fig. 5). Therefore, this IEM apparently generated non-infectious HDV virions. There could be more than one reason for such a profound impact on the infectivity. Based on this IEM's location on the right-hand side of MAL, which spans the positions (pos.) 150–172 and bears only four of the tested IEMs, and resides apart from the two sub-loops and closer to transmembrane domain III (TMD III), it is uncertain whether R169 residue could be directly involved in the formation of major structural elements of MAL (Fig. 8B). However, it is possible that R(169)P change, via introducing a bend or a turn in the amino acid chain, may have a long-range effect either on the overall structure of MAL and/or on the structure of TMD III, and/or on TMD III positioning in the membrane (Fig. 8B), which could lead to alteration of HBsAg functioning. It was also previously noted that R(169)P greatly reduced the amounts of the secreted envelope proteins, while not affecting the levels of intracellular HBsAg, which indicated a specific defect in the egress of the produced envelope proteins (10, 11). Our observations were also consistent with another report suggesting that the putative amphipathic alpha helix spanning positions 156–169 could play a critical role in the morphogenesis of the SVPs (39). Taken together, our results and the findings from other labs further suggest that there is likely a defect in the HBsAg-host interactions for the IEM R(169)P, which could critically impair the secretion of HDV virions (for the HBV genotypes B and D [Fig. 2]) and affect viral entry as well, assuming that the same amino acid involved in egress also contributes to virus-host interaction during entry. Furthermore, our analysis, based on treatment with the benzonase nuclease, indicated that the R(169)P-containing HBsAg formed HDV virions, about half of which were defective in terms of protecting the integrity of the virion-bound HDV G RNA (Fig. 4), that also very likely contributed to the observed loss of the infectivity.

The group of the remaining 12 IEMs that dramatically reduced the infectivity of the HDV virions includes the following mutations: G(119)E, C(121)Y, R(122)I, T(123)N, C(124)R, S(132)F, C(137)R, C(138)Y, K(141)E, P(142)S, N(146)S, and C(149)R. These mutations in HBsAg reduced the levels of the infection-generated intracellular HDV G RNA within the range from 7.8- to 259.6-fold. At the same time, the relative percentage of the infected cells was reduced for these IEMs in the range from about 3.7- to 51.3-fold (Fig. 8A). Corresponding images for the HepG2-NTCP cells infected by HDV virions bearing these IEMs are presented in Fig. 5. The observed numbers of HDV-infected cells for the above-mentioned IEMs were greatly decreased compared to the cells infected with HDV enveloped with wt HBsAg (Fig. 5). These 12 amino acid residues, which were most critically affected by the IEMs in terms of the HDV infectivity, belong to the two distinct stretches of amino acids in the MAL. The first stretch spans the pos. from 119 to 124 located at the top of the first sub-loop of the MAL, which is formed by C107–C138 S–S bond (Fig. 8B). The second stretch covers the pos. 137–149, which are situated in the very small second sub-loop of the MAL formed by C137–C149 S–S bond (Fig. 8B). Within the boundaries of the second stretch, only pos. T140, S143, D144, and G145 were functionally quite tolerable to the tested IEMs. The only one IEM S(132)F, greatly reducing the infectivity, is located outside of the above-mentioned two stretches. Its position is in the middle of the right-hand side of the first sub-loop of the MAL (Fig. 8B).

It needs to be noted that, very interestingly, the mutations G(119)E, C(121)Y, R(122)I, C(124)R, and K(141)E resulted in a more pronounced effect on the levels of HDV G RNA, while affecting the relative percentage of the infected cells to a considerably lesser extent (Fig. 8A). For these IEMs, the values of the relative percentage of the infected cells were 5.44- to 8.77-fold higher than the corresponding values of the levels of intracellular HDV G RNA, which was not the case for the other 7/12 mutants. Thus, for example, for mutation C(121)Y, the levels of HDV G RNA were 1.26%, while the relative percentage of the infected cells was 11.06%. For the IEM R(122)I, the levels of HDV G RNA were 0.62% and the relative percentage of the infected cells was 5.01%. Similarly, for C(124)R, the levels of HDV G RNA amounted to 1.56%, but the relative percentage of the infected cells was 11.24%, etc. (Fig. 8A). One possible interpretation of these observations is that the five above-mentioned IEMs more substantially influenced the intracellular post-entry trafficking of HDV virions, which resulted in a profound reduction of the amounts of HDV RNPs that reached the nucleus and started HDV genome replication. While at the same time, the relative percentage of the infected cells was affected to a markedly lower extent, which could be consistent with the notion that the same IEMs did not have such a substantial effect on the HDV attachment and entry. Such interpretation is consistent with the belief that MAL residues could likely regulate both the number of HDV-infected cells and the number of HDV genomes (in the context of HDV RNPs) that reached the nuclear replication sites and started the HDV genome replication process. Furthermore, the finding that certain IEMs resulted in considerable differences in the extent of the reduction of (i) the levels of HDV G RNA versus (ii) the relative percentage of the infected cells suggests an interesting possibility that the MAL could bear the residues mostly involved in the attachment/entry, as well as the other residues primarily regulating the post-entry trafficking (until the uncoating step). This theory is in agreement with the opinion that, being on the outer surface of the virions, the MAL is most certainly involved in the regulation of attachment/entry and post-entry trafficking of the virions. Since the MAL is not directly involved in the binding of the viral cargo of HDV (i.e., HDV RNP) or HBV (nucleocapsid with its contents), it may possibly only indirectly influence the uncoating process. Therefore, follow-up studies investigating in detail and more directly the involvement of the MAL residues in the regulation of attachment/entry and the intracellular trafficking of the virions, as well as the MAL’s potential to affect the virions’ uncoating, are warranted.

In addition, it needs to be mentioned that IEM G(159)C did not affect much HDV assembly and HDV infectivity (Fig. 2, 5, and 8), while it was creating an additional Cys residue that potentially could be engaged in the formation of new S–S bonds and thus could alter the structural organization of the MAL. One explanation here is that the structure of the MAL, which was chosen to be used in this work (Fig. 1), more closely reflects the actual MAL structure *in vivo*, in which all Cys residues were involved in the formation of the S–S bonds, and there were no vacant Cys residues that could, in theory, interact with new Cys created in pos. 159 by the above-mentioned IEM, while there are other reported alternative MAL structures even employing different lengths of the MAL and proposing different structural architecture of this critical element of HBsAg (5, 10, 20–24, 40).

## DISCUSSION

One of the outcomes of HBV-host interactions is the emergence of the HBV immune escape mutants bearing amino acid substitutions in the MAL (Fig. 1, [8–22, 40]), which is a structural element that is present in all three HBV envelope proteins, large (L), middle (M), and small (S). The MAL not only contains the antigenic sites recognized by anti-HBsAg antibodies but also plays a critical role in facilitating the assembly and infectivity of the HBV and HDV virions (1, 3, 6, 7, 23–25, 41). Since the HBV envelope proteins mediate the formation of both HBV and HDV virions (1, 3, 6, 7, 41), it is important to evaluate how IEMs affect the assembly and infectivity of HDV, and thus to examine how anti-HBV immune response influences the life cycle of this natural sub-viral agent of HBV. The relationship between IEMs of HBV and HDV life cycle has not been extensively studied previously. This study is the first comprehensive analysis of the regulation of the HDV assembly and infectivity by the IEMs of HBV, which examined a considerable number of natural IEMs (Table 1).

Thirty-five natural immune escape HBV mutations (Table 1; Fig. 1) have been examined for their effects on the assembly and infectivity of HDV. We found that all tested IEMs were permissive for HDV assembly and secretion. Twenty-eight tested IEMs displayed no considerable effect on HDV assembly levels. For these IEMs, the observed decrease or increase (if any) in the measured levels of secreted HDV virions was less than 3-fold (Fig. 2A). Some of the IEMs were studied before for their effects on HDV properties. Thus, similar to the previous report (23), in our settings, P(120)S did not have any significant effect on HDV assembly. The changes K(141)E and G(145)R were reported to apparently reduce the yield of HDV virions to a certain extent (23). While we observed reduced HDV assembly by about ~4-fold for G(145)R in the context of the HBV genotypes B and C, the K(141)E change did not cause any considerable reduction of the amounts of secreted HDV virions (Fig. 2).

Only seven out of thirty-five tested IEMs reduced the yield of secreted HDV virions by more than 3-fold (Fig. 2). Only C(138)Y mediated a considerable decrease in HDV assembly in the context of all three tested HBV genotypes, B, C, and D. The mutations G(145)R, C(149)R, and R(169)P decreased HDV assembly levels for two out of three tested HBV genotypes. The remaining 3/7 IEMs, I(110)M, P(127)R, and N(146)S, substantially affected the levels of secreted HDV only in the context of one out of three tested HBV genotypes (Fig. 2). Therefore, there were observed certain HBV genotype-specific differences among the examined IEMs, which suggested that while the envelope proteins of different HBV genotypes facilitate basically the same functions, including mediating the formation and secretion of the HDV virions, they apparently do it via not exactly the same mechanisms. It is interesting to note that such differences in terms of tolerating IEMs effects to a different extent may contribute to the preference in the survival rates for one HBV genotype over the other *in vivo*. In addition, our further analysis was consistent with the interpretation that the tested IEMs did not compromise the integrity of the secreted virion-bound HDV G RNA, with one exception. Only the IEM R(169)P apparently altered the ability of HBsAg to protect HDV G RNA inside the virions (Fig. 4).

We also assayed the yield of secreted HBsAg (Fig. 3) in relation to the observed effects of IEMs on the HDV assembly levels (Fig. 2). As described above in the Results, we measured diverse levels of secreted HBsAg, with some effects appearing to be specific to the tested HBV genotypes (Fig. 3). Since, for only 7/30 IEMs, substantially decreased HDV assembly levels were found, it should be noted that in the remaining 23/30 IEM cases, there were enough functional HBsAg produced to ensure considerable yields of secreted HDV virions. The yields of secreted HDV virions observed in those cases differed less than 3-fold from the yields observed for the HDV virions coated with the corresponding wt HBsAg (Fig. 2), regardless of the measured HBsAg levels (Fig. 3). Our data were also consistent with the previous observations demonstrating that frequently there was no strict correlation between the levels of secreted HBsAg and secreted HDV virions, and the yield of HDV virions often could not be explained by the efficiency of HBsAg secretion (23, 24, 27, 30, 31). However, having considered the levels of secreted HBsAg, we came to the realization that for the mutation I(110)M, the main factor contributing to decreased HDV assembly was altered by the IEMs functional ability of HBsAg to promote the formation and/or secretion of HDV virions, and not the efficiency of HBsAg secretion. For the P(127)R and N(146)S, the HBsAg functioning affected by these IEMs was likely a substantial contributing factor toward the reduced levels of HDV assembly (Fig. 2 and 3). The I(110)M and P(127)R are located in the first sub-loop of the MAL, while the third one, in pos. 146, resides in the second sub-loop (Fig. 2B), and it is possible that these three residues—I(110), P(127), and N(146)—are involved in critical interactions with host factors during the formation and secretion of HDV virions. Therefore, further follow-up investigation into the involvement of these three amino acid residues of the MAL, I(110), P(127), and N(146), in interactions with host factors during the formation of HDV virions and their secretion is warranted. In the case of the mutations R(169)P and G(145)R, the major impact in terms of the reduction of HDV assembly levels was most likely mediated by a considerably reduced yield of secreted HBsAg (Fig. 2 and 3). At the same time, since, as mentioned above, the IEM R(169)P apparently facilitated the production of defective HDV virions with compromised capacity to protect the virion-bound HDV G RNA (Fig. 4), we could speculate that this IEM-mediated altered functioning of HBsAg could potentially contribute to some extent to the reduced HDV assembly as well. For the C(149)R and C(138)Y, the situation appeared to be more complex, and while the reduction in HBsAg levels could be a considerable contributor toward decreased HDV assembly, some functional alterations in HBsAg properties could also have an impact on the yield of secreted HDV virions (Fig. 2 and 3). Therefore, the analysis in the follow-up studies should look into more details of how the two above-mentioned reasons could have contributed to diminished levels of secreted HDV virions mediated by IEMs. It was also noted that certain IEMs affect the secretion of HBsAg in the HBV genotype-specific manner, which again indicated that regardless of the notion that the envelope proteins of different HBV genotypes basically are expected to facilitate the same functions, the exact mechanisms of how they act are not exactly identical, which allowed some of the HBV genotypes to better tolerate the IEMs-mediated effects on the HBsAg properties, which in-turn could reflect the outcomes of the *in vivo* competition between different HBV genotypes in the same host. In addition, it is worth mentioning that all the seven positions I110, P127, C138, G145, N146, C149, and R169 were previously reported as critically important for the regulation of the secretion of HBV virions as well (10–12). Furthermore, while the IEMs, I(110)M, C(149)R, C(138)Y, G(145)R, N(146)S, and R(169)P, were able to severely impair the secretion of HBV virions, another mutant P(127)S, which was not an IEM, also caused considerably reduced HBV assembly. In fact, for the IEMs I(110)M, C(138)Y, G(145)R, N(146)S, C(149)R, and R(169)P secreted HBV virion-bound DNA was barely detectable or even undetectable (10–12). Therefore, the effects of the above-mentioned IEMs were similar in terms of reducing the levels of secreted HDV and HBV virions, while their impact on HBV secretion seemed to be more pronounced. However, there were also some differences in the effects of the IEMs on HDV versus HBV. Thus, the IEMs 114R (i.e., in the original study (12), it was T(114)R, while in the current study, the mutation was S(114)R), T(118)K, G(119)E, and K(141)E substantially diminished the yield of the secreted virions for HBV (10–13, 15), but not for HDV (Fig. 2; Table 1), which was consistent with the notion that HDV and HBV have different assembly mechanisms (1, 41).

We further found that IEMs had a much more pronounced influence on the infectivity of HDV virions than on their assembly. Thirteen out of thirty-five tested IEMs (Fig. 8) very significantly decreased HDV infectivity. One of these IEMs, R(169)P, completely abolished the infectivity of HDV virions, resulting in undetectable levels of HDV G RNA and the absence of detectable HDV-infected cells (Fig. 8). The profound reduction of HDV infectivity observed for the above-mentioned 13 IEMs resulted in a great reduction of both (i) the levels of the HDV RNA genomes accumulated in the infected cells post-infection (Fig. 8A), and (ii) the percentage of the HDV-infected cells (Fig. 5, 6, and 8). The reduction of the infectivity of HDV virions should lead to suppression of the viral spread and super-infection *in vivo*, which is expected to facilitate the decrease of the size of the HDV reservoir in the infected livers.

**Fig 9:**
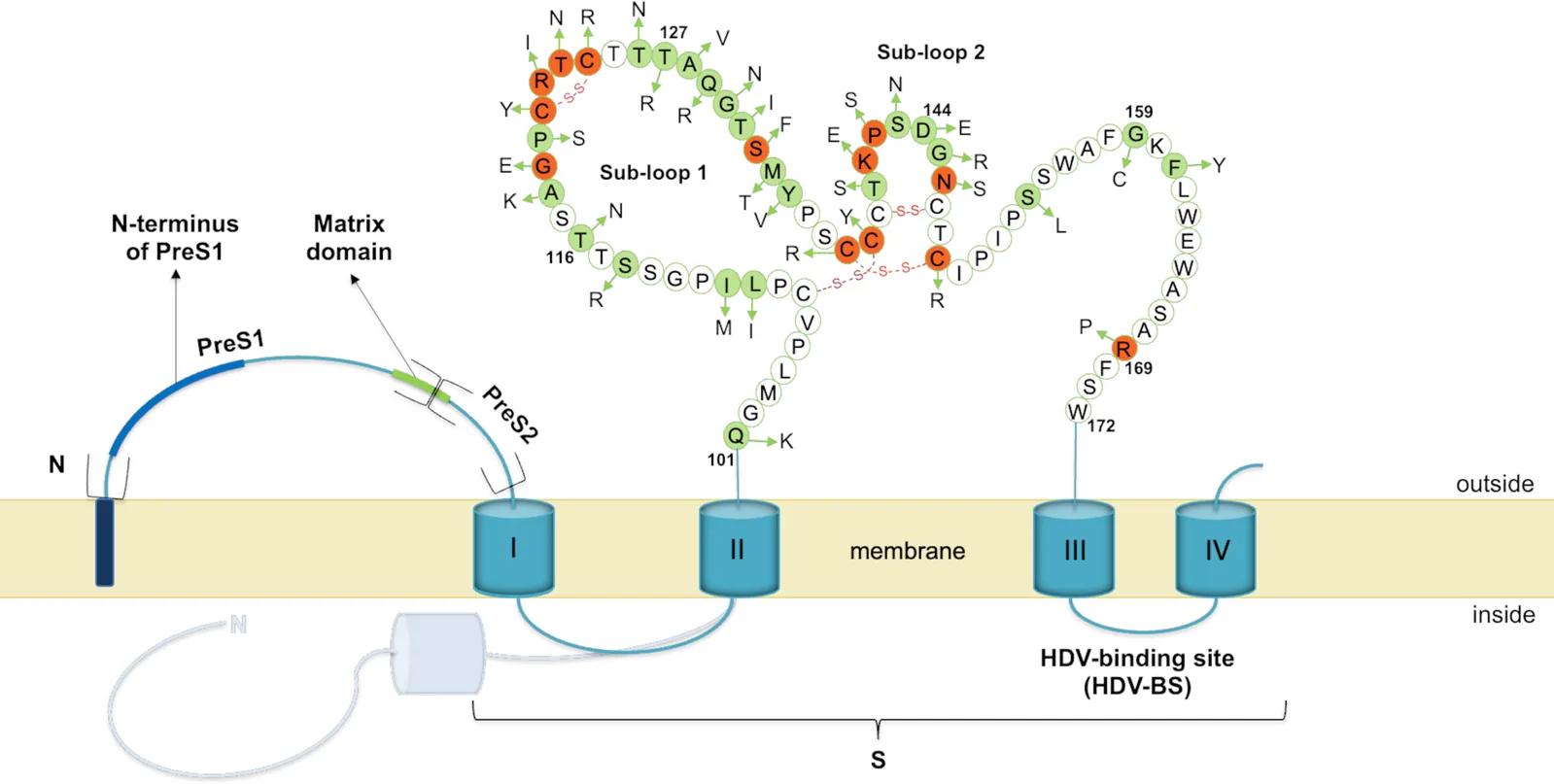
Proposed modified structure of the major antigenic loop. The figure depicts the modified structure of the MAL with the second sub-loop protruding outward (not inward as it was in the initial representation of the MAL structure [see Fig. 1 to 3, and 8] [5]) in order to reflect our findings suggesting the accessibility of the key amino acids, which participate in the regulation of HDV virion infectivity, for interacting with cellular (host) factors during attachment/entry and/or subsequent post-entry intracellular trafficking of the virions up to the point of uncoating (i.e., losing the envelope consisting mostly of HBV envelope proteins). The IEMs greatly affecting HDV infectivity are marked in orange. The rest of the IEMs are shown in light green, while the positions not analyzed in this study are represented by uncolored circles. The presented MAL sequence reflects genotype D of HBV. The structural elements of the L envelope protein are shown as in Fig. 1.

The remaining 12 IEMs most profoundly affecting infectivity of HDV virions (i.e., both the levels of the infection-generated HDV G RNA and the relative percentage of the infected cells), G(119)E, C(121)Y, R(122)I, T(123)N, C(124)R, S(132)F, C(137)R, C(138)Y, K(141)E, P(142)S, N(146)S, and C(149)R, are located in the two sub-loops of the MAL (Fig. 8). The first six IEMs are located within the area covering pos. 119–136 and are situated at the top and at the right-hand side of the first sub-loop of MAL formed by the S–S bond. The rest of the amino acids, which are critical for HDV infectivity, are localized within the second (small) sub-loop of the MAL spanning the area of pos. 137–149 (Fig. 8B). The structural elements of the MAL bearing those important regulators of HDV infectivity are likely to be involved in critical virus-cell interactions during attachment and entry and/or post-entry intracellular trafficking because the MAL is exposed on the outside of the virions. Our results support a clover-like structure of the MAL with both sub-loops exposed and available for virus-cell interactions, and with all cysteines engaged in the formation of disulfide bonds, i.e., without vacant Cys residues (Fig. 9). The absence of vacant cysteines in the MAL was also suggested by previous studies (34, 42, 43). One previous study also noted that the domain of the MAL covering pos. 119–128 was critical for the infectivity of HDV virions *in vitro* (24), and a later report included a considerable number of residues in the area of pos. 137–156 (i.e., pos. 137–139, 141, 142, 146–150, 153, and 156) as major regulators of HDV infectivity (23), which is in tune with the outcomes of our examination of the effects of natural IEMs of the MAL on HDV infectivity (Fig. 8).

Interestingly, the comparison of the levels of HDV G RNA and the corresponding relative percentages of HDV-infected cells for the above-mentioned IEMs reducing HDV infectivity, for which the relative percentages of the HDV-infected cells were reduced to a considerably lesser extent as compared to the reduction of the levels of intracellular HDV G RNA [i.e., G(119)E, C(121)Y, R(122)I, C(124)R, and K(141)E] (Fig. 8A), allowed us to interpret that the amino acid residues of MAL could likely participate in regulating both the virions’ attachment/entry and certain steps of post-entry trafficking of HDV virions up until the point when the virions undergo uncoating (loss of the envelope coating the virions). Our previous work led to the interpretation that, apparently, the HBV envelope proteins *per se* determined the number of HDV-infected cells and the number of HDV genomes (in the context of HDV RNPs) that reached the nucleus and started HDV RNA genome replication (27). In agreement with this interpretation (27), by comparison of the levels of HDV G RNA to the corresponding relative percentages of the HDV-infected cells, we could infer that certain different residues of the MAL could possibly primarily facilitate the attachment/entry of the virions, while the others could more likely be governing the post-entry trafficking of the virions. Thus, the mutations G(119)E, C(121)Y, R(122)I, C(124)R, and K(141)E, which had more pronounced effects on the levels of HDV G RNA as compared to the relative percentage of the infected cells, apparently could be more primarily involved in the regulation of the post-entry trafficking of the virions, and at the same time could have much lesser involvement in the attachment and entry of the virions (that would be expected to be reflected by the percentage of the infected cells) (Fig. 5 and 8A). Therefore, further follow-up studies that will use more direct experimental approaches and examine in more detail the functioning of the amino acids that could primarily mediate either of the two above-mentioned functions important for the infection (i.e., attachment/entry or post-entry trafficking) are warranted. It needs to be acknowledged that, however, productive infection only takes place when the MAL works in concert with the PreS1 domain that mediates critical sequence-specific interactions with the NTCP, the specific HBV receptor (1–4, 25). The above-described findings further advanced our understanding of the aspects of the mechanism by which MAL residues function during the infection of susceptible cells.

**Fig 10:**
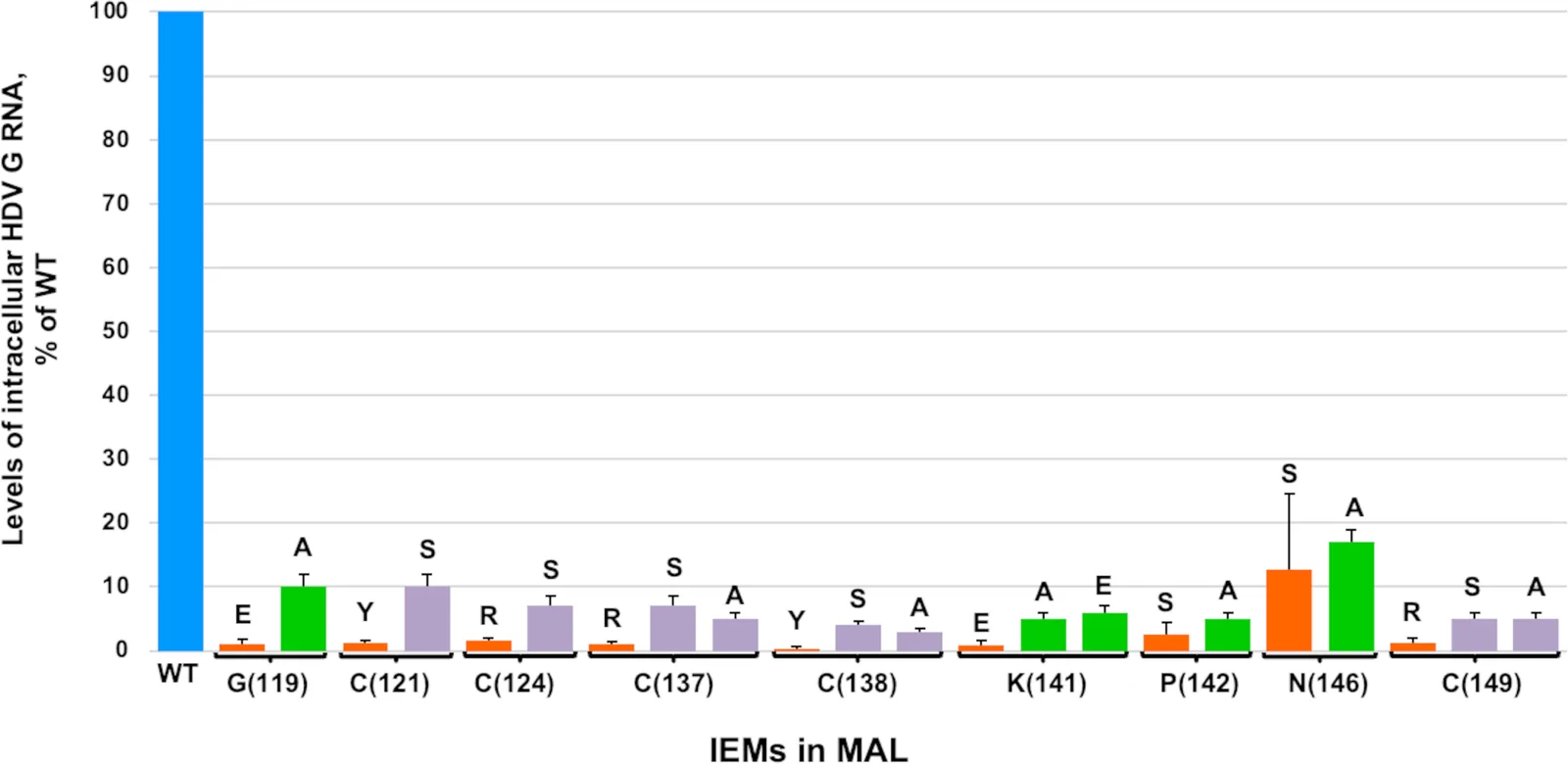
Residues in the major antigenic loop important for the infectivity of HDV virions, for which the reduction of infectivity was observed as a result of all known mutations. The figure depicts the amino acid residues in the MAL that are important for the infectivity of the HDV virions and that behave as invariant residues because mutating them to different amino acids results in the reduction of the infectivity of HDV. Because others did not analyze the percentage of the HDV-infected cells, the only measure of infectivity in their work was the analysis of the accumulation of the infection-generated HDV genomic RNA in infected cells, which is analogous to our measurements of the levels of HDV G RNA in infected HepG2-NTCP cells, and also is expressed as the percentage of the value obtained for the not mutated wt control HBsAg (that was used as 100% value). Thus, the figure only reflects the accumulation of the infection-generated HDV genomes in infected cells relatively to that obtained for HDV virions coated with wt (not mutated) HBsAg. The figure takes into consideration our findings (orange bars) and the findings reported by others (23 [green bars], 34 [light purple bars]). The amino acid to which the original residue was changed is indicated above each bar. The presented data are for genotype D of HBV.

**Fig 11:**
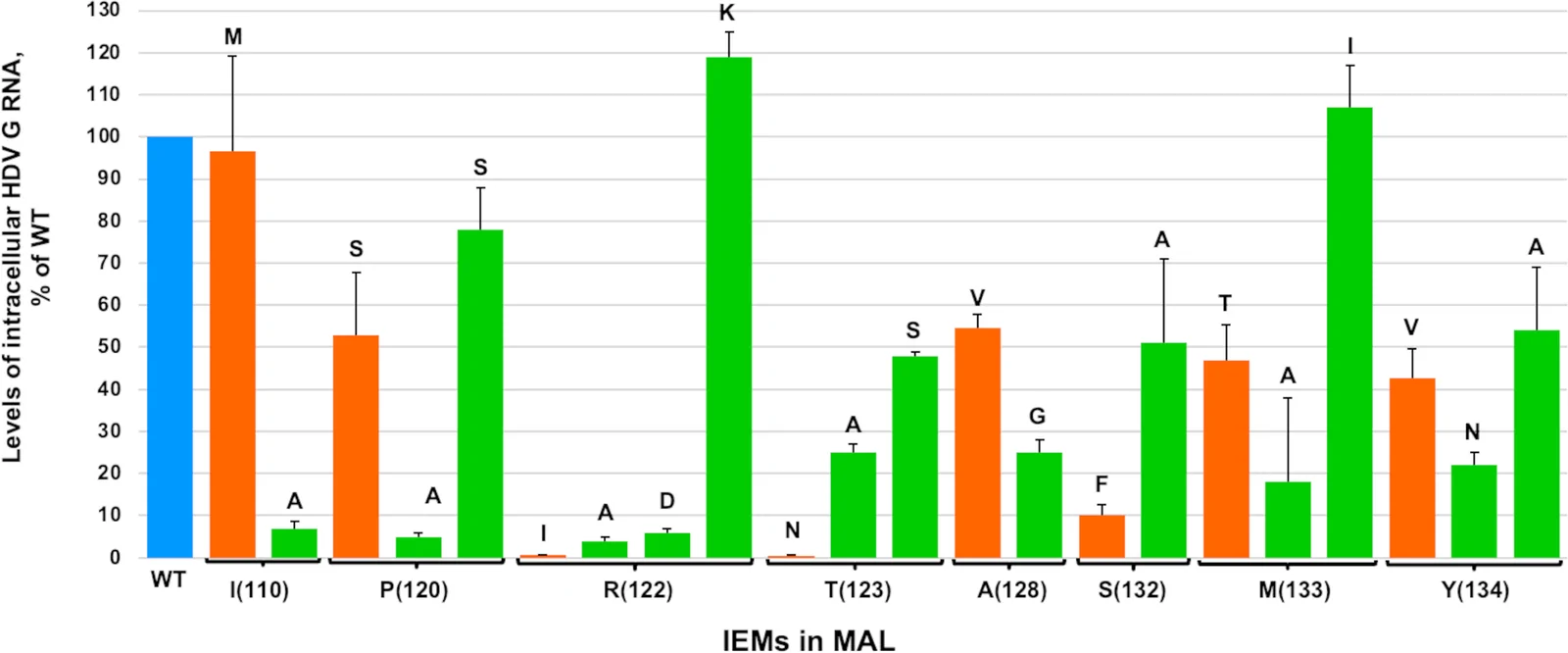
Residues in the major antigenic loop important for the infectivity of HDV virions, for which the reduction of the infectivity was observed only if they were mutated to a particular amino acid. The figure depicts the amino acid residues in the MAL that are important for the infectivity of the HDV virions and that do not behave as invariant residues, because changing them to different amino acids results in the reduction of the infectivity of HDV only in some cases, and not in others. This demonstrates that some substitutions of the amino acid residue(s) can be tolerated at the particular positions in the MAL with maintaining the function mediating the HDV infectivity. Because others have not analyzed the percentage of the HDV-infected cells, the only measure of infectivity in their work was the analysis of the accumulation of the infection-generated HDV G RNA in infected cells, which is analogous to our measurements of the levels of HDV G RNA in the infected cells, and also is expressed as the percentage relatively to the value obtained for wt control HBsAg (that was used as 100% value). Similarly to Fig. 10, this figure therefore only reflects the accumulation of the infection-generated HDV genomes in infected cells relatively to that obtained for HDV virions coated with wt (not mutated) HBsAg. The figure considers our findings (orange bars) and the findings published by others (green bars) (23). The amino acid, to which the original residue was changed, is indicated above each bar. The presented data are for genotype D of HBV.

In addition, it needs to be emphasized that there were apparently two different subsets of the amino acid residues in MAL in terms of the influence of the mutations on the functioning of these residues. The first one represented the residues, mutating of which to different amino acids at the same position resulted in the same functional effect (Fig. 10). These amino acid residues therefore behave as invariant amino acids in the MAL. The second subset included the residues, mutating of which to only certain amino acids resulted in a functional effect, which was not the case for the other amino acid substitutions at the same position (Fig. 11).

The five Cys residues belong to the first subset (Fig. 10). We found that IEMs C(121)Y, C(124)R, C(137)R, C(138)Y, and C(149)R greatly reduced HDV infectivity (Fig. 8). Similarly, others reported that changes C(121)S, C(124)S, C(137)S and C(137)A, C(138)S and C(138)A, C(149)S and C(149)A also diminished the levels of the intracellular HDV genomes generated by the infection (23, 34). It has also been demonstrated by using thiol-disulfide exchange inhibitors in order to disrupt the S–S bonds in the MAL that the correct functioning of the conformational structural elements in the MAL, formation of which was facilitated by the creation of disulfide bonds between certain Cys residues, was vital for ensuring the infectivity of the formed HDV virions (34). Importantly, the previous studies were also able to demonstrate that seven cysteines at positions 121, 124, 137, 138, 139, 147, and 149 in the MAL, which included the above-mentioned five positions, were critical for the infectivity function of the assembled virions (23, 24, 34). In addition, based on the above-mentioned previous studies, it was suggested that the MAL “is potentially active at two steps of the viral entry pathway: (i) the ‘a’ determinant being involved in a binding function and (ii) the intermolecular disulfide network controlling the envelope disassembly process” (34). This is consistent with the above-mentioned interpretations of the outcomes of our experiments as well. However, it needs to be noted that, as described above, we analyzed both the intracellular levels of the infection-generated HDV G RNA and the percentage of the infected cells. While no other lab has examined the influence of the mutations in the MAL on the percentage of the HDV-infected cells.

We also considered several recent publications dedicated to resolving the structural aspects of the formation of the SVPs assembled out of HBsAg molecules (44–46). The studies seemed to be in agreement that the MAL, in which the analyzed IEMs resided, is protruded on the outside of the particles, which is apparently consistent with its potential functions in terms of attachment/entry, post-entry intracellular trafficking, and binding with anti-HBsAg antibodies. The studies recognized the importance of the MAL structure, which was supported by the formation of the intramolecular disulfide bonds between the Cys residues, and also emphasized the participation of the MAL sequences in the intermolecular interactions between different HBsAg molecules, which are important for the formation of the SVPs and the virions. While two of the above-mentioned structural reports (44, 45) could not perform fine modeling of the structural elements of the MAL because of the low resolution, the third report (46) suggested the formation of the intramolecular S–S bonds between Cys121 and Cys124, Cys137 and Cys149, and also between Cys139 and Cys147, which is completely in agreement with the representation of these three S–S bonds in Fig. 1 to 3, and 8. At the same time, it has been suggested that Cys107 is involved in the formation of the intermolecular bond with the corresponding Cys107 on the other HBsAg molecule (46). This all is consistent with the critical importance of the cysteines 121, 124, 137, and 149, which are shown as engaged in the formation of intramolecular S–S bonds in Fig. 1 to 3, 8, and 9, for the infectivity of the HBsAg-coated HDV virions (Fig. 8B). Figures 1 to 3, 8B, and 9 also show the intramolecular S–S bond between Cys139 and Cys147, while there were no IEMs reported at these sites and at position 107 (8–22), and thus no IEMs were tested at positions 107, 139, and 147 in the current study (Table 1). The structure of the MAL shown in Fig. 1 suggests the absence of vacant Cys residues, i.e., cysteines not engaged in forming S–S bonds. As mentioned above, our data also support the absence of Cys residues not participating in the formation of S–S bonds (Fig. 9). Furthermore, the absence of vacant Cys residues in the MAL not engaged in S–S bond formation was also supported by previous reports (34, 42, 43). Therefore, considering our data and the above-mentioned report (46), follow-up studies should try to find out (i) if at least a portion of Cys107 residues could be engaged in S–S bond formation with Cys138 of the same HBsAg molecule (as shown in Fig. 1 and 9) as well (and not only form intermolecular S–S bonds [46]), and (ii) if a portion of Cys138 residues also forms S–S bonds with Cys107 within the same molecule, while the rest of Cys138 residues may be involved in the formation of disulfide bonds with Cys138 residues on other HBsAg molecules. Both these scenarios will be consistent with our findings demonstrating that Cys138 is critical for facilitating HDV infectivity (Fig. 8B). In addition, the IEM G(119)E (Fig. 8) and the change G(119)A (23) also both greatly decreased HDV infectivity. The same was the case for other changes like IEM K(141)E (Fig. 8) and K(141)A (23), IEM P(142)S (Fig. 8) and P(142)A (23), and IEM N(146)S (Fig. 8) and N(146)A (23). The above-mentioned mutations are displayed and compared in Fig. 10.

A number of the positions in the MAL belong to the second above-mentioned subset. The influence of different amino acid substitutions at the same positions for these residues is summarized in Fig. 11. For example, IEM I(110)M had little effect on HDV infectivity (Fig. 8A), while I(110)A greatly reduced infectivity ([23], Fig. 11). The IEM R(122)I dramatically inhibited HDV infectivity (Fig. 8). The changes R(122)A and R(122)D had a similar effect, but R(122)K practically had no effect on the infectivity ([23], Fig. 11). The latter was expected because wt sequences of genotypes B and C bear K at position 122 (Table 1). This indicates that the presence of a positive charge at this position is critical for MAL functioning. The IEM P(120)S did not result in reducing HDV infectivity (Fig. 8A, [23]), while the substitution P(120)A significantly inhibited HDV infectivity ([23], Fig. 11). The IEM T(123)N dramatically decreased HDV infectivity (Fig. 8), while the change T(123)A reduced infectivity only by 4-fold, and T(123)S only decreased it by ~2-fold ([23], Fig. 11). The IEM S(132)F had a major down-regulating effect on HDV infectivity (Fig. 8), but the change S(132)A did not ([23], Fig. 11). The IEM A(128)V caused only a slight reduction of infectivity to approximately 55% (Fig. 8A), while the change A(128)G resulted in an infectivity decrease by 4-fold (Fig. 11, [23]). The IEM M(133)T decreased infectivity by about 2-fold only (Fig. 8A), but the mutation M(133)A reduced it by about 5.6-fold, while at the same time, the change M(133)I had no significant effect (Fig. 11, [23]). The IEM Y(134)V only slightly decreased infectivity by about 2-fold (Fig. 8A), and the change Y(134)A had practically the same effect, but Y(134)N led to about 5-fold infectivity reduction ([23], Fig. 11).

Finally, only four tested IEMs, C(138)Y, N(146)S (which is the major glycosylation site in the MAL [30]), C(149)R, and R(169)P, had considerable effects on both the assembly and infectivity of HDV (Fig. 2, 5, and 8). In addition, our results were consistent with the previous report (23) showing that the change K(141)E greatly reduced HDV infectivity, while the other two IEMs, P(120)S and G(145)R, did not. These three above-mentioned IEMs thus served as internal references in our study (Table 1).

In summary, this is the first study that conducted a systematic and detailed analysis of a large collection of the natural HBV IEMs located in the MAL of the HBV envelope proteins in terms of their influence on the assembly and infectivity of HDV, which is the sub-viral agent of HBV and uses HBV envelope proteins to form its own virions and to infect the susceptible cells using the HBV receptor (1, 3, 4). Overall, our findings demonstrated that IEMs can exert considerable inhibitory influence on both HDV assembly and infectivity. The IEMs’ effects on HDV infectivity were much more pronounced than their effects on HDV assembly. It is also important to realize that if the IEMs that are detrimental for HDV assembly and/or infectivity are present on the majority of the HBsAg molecules (regardless of their origin, whether they are produced by HBV genome replication or translated from the 5′-HBV-human-3′ RNAs, which were transcribed from integrated HBV DNA independently of HBV genome replication [47]), this could considerably reduce the efficiency of HDV spread and super-infection *in vivo*, which are the critical determinants of the establishment and maintenance of chronic HDV infection (28, 36, 47–49). At the same time, the suppression of HDV spread and super-infection could lead to a significant reduction in the size of the HDV reservoir in the infected livers and thus to substantial suppression of HDV infection, which in turn could possibly result in reducing HDV-related liver pathogenesis and HDV-associated liver disease as well. These findings therefore showed that anti-HBV immune response can considerably down-regulate concomitant HDV infection *in vivo*. Furthermore, the generated data also advanced our understanding of the complex intermicrobial HBV-HDV relationship by demonstrating how the natural IEMs in HBsAg regulate HDV assembly and infectivity, which can affect the course of HDV infection *in vivo* and also regulate the persistence of HDV by shrinking its liver reservoir size.

## MATERIALS AND METHODS

### Making the constructs bearing natural HBV immune escape mutations in HBsAg

The natural HBV IEMs examined in this study are listed in Table 1. All 35 IEMs were located in the MAL. To make the mutations in HBsAg of three HBV genotypes, we used three previously described constructs, each expressing the L, M, and S envelope proteins of HBV: pNF-HBV-LMS-B2 (for genotype B of HBV, the accession number for this variant of HBV was AB205119), pNF-HBV-LMS-C5 (for genotype C, the accession number AF411411), which is a natural M-minus mutant and expresses only L and S envelope proteins, and does not express M envelope protein, and pNF-HBV-LMS-D1 (for genotype D, the accession number AB205127) (27). The plasmids pNF-HBV-LMS-C5 and pNF-HBV-LMS-D1 expressed the HBV envelope proteins not bearing any IEMs, and thus they were used as vectors expressing wt HBsAg of the corresponding HBV genotypes. The two changes were introduced into the MAL sequence in the plasmid pNF-HBV-LMS-B2: M(110)I and Y(161)F (the numbering is for the small envelope protein of HBV) to eliminate two IEMs that the natural HBV variant, the sequence of which was used to make pNF-HBV-LMS-B2, contained. The resulting vector was named pNF-HBV-LMS-B2wt and used as the construct expressing wt HBsAg of HBV genotype B and not bearing any IEMs. Next, the IEMs were introduced into the constructs pNF-HBV-LMS-B2wt, pNF-HBV-LMS-C5, and pNF-HBV-LMS-D1 using the site-directed mutagenesis kit QuickChange II (Agilent). For each HBV genotype, we made 35 different HBsAg-expressing vectors, each bearing a single IEM in the MAL (Table 1).

### Transfection to produce HDV virions

In order to assemble HDV virions enveloped with HBsAg of different HBV genotypes (B, C, or D) and not bearing (wt) or bearing IEMs, Huh7 cells were co-transfected (using a 1:1 ratio) with the plasmid pSVLD3 initiating HDV RNA genome replication and a plasmid expressing HBsAg of a particular HBV genotype with or without IEM in it using Lipofectamine 2000 (Invitrogen) according to the instructions of the manufacturer (27, 28). For the analysis of the levels of secreted HDV virions, the media from the transfected cells were harvested at day 9 post-transfection and analyzed for the copy number content of HDV genomic RNA using the specific RT-qPCR procedure (27–29). The same media were also used for the analysis of secreted HBsAg using ELISA (Abbexa).

### RNA isolation for RT-qPCR analysis

Total RNA was isolated from the media of transfected Huh7 cells or from infected HepG2-NTCP cells by using TRI Reagent (Molecular Research Center) in accordance with the instructions of the manufacturer. The isolated total RNA was treated with 7 U of Turbo DNase (Fisher) for 1 h at 37°C. DNase-treated RNA samples were re-extracted with TRI reagent and then used for synthesis of cDNA using LunaScript RT SuperMix kit (New England Biolabs) (50, 51).

### Analysis of the levels of secreted and intracellular HDV genomic RNA using RT-qPCR

The HDV-specific qPCR amplifying the sequences of HDV genomic RNA employed the forward primer (312-GGACCCCTTCAGCGAACA-329), the reverse primer (393-CCTAGCATCTCCTCCTATCGCTAT-360), the TaqMan probe [5′-FAM(6-carboxyfluorescein)-332-AGGCGCTTCGAGCGGTAGGAGTAAGA-357-3′-BHQ-1 (Black Hole Quencher-1) (the numbering of HDV positions is according to Kuo et al. [26])], and TaqMan Gene Expression Master Mix (Fisher). The above-mentioned reverse primer was also used for cDNA synthesis. The copy numbers of HDV genomic RNA (HDV G RNA) were quantified using 10-fold dilutions of *in vitro*-transcribed and gel-purified unit-length HDV G RNA standard (in the range from 200 to 20,000,000 GE of HDV) (29, 31, 52).

### Analysis of secreted HDV genomic RNA using the benzonase nuclease treatment

The analysis was basically done as described previously (36). Briefly, the media were collected from transfected Huh7 cells at day 9 post-transfection and briefly centrifuged to remove cell debris. The clarified supernatant was treated with Benzonase nuclease (Millipore) at a final concentration of 2 U/µL. The reaction mixture was also supplemented with 50 mM Tris-HCl (pH 8.0), 1 mM MgCl_2_, and 100 mg/µL BSA. The reaction was conducted at 37°C for 24 h. The control reactions were done in the absence of the benzonase. After the treatment, the samples were subjected to RNA extraction using TRI reagent according to the procedure described above, and the amounts of HDV G RNA were quantified by RT-qPCR. The levels of the HDV G RNA recovered after the benzonase treatment were shown as the percentage of the levels of HDV G RNA recovered from control (no benzonase) reactions. At the same time, the functional activity of the benzonase was verified by treating HDV G RNA isolated from the HDV virions, which resulted in predominant destruction of the “unprotected” isolated HDV G RNA (not shown).

### Infection of HepG2-NTCP cells with HDV

The HDV virions were produced by the co-transfection approach in Huh7 cells as described above (27, 28). For the analysis of infectivity, the IEMs in the context of HBV genotype D were analyzed. In this case, the media from co-transfected Huh7 cells were collected from day 7 to day 10 post-transfection, and then concentrated using polyethylene glycol (PEG) (Fisher) as previously described (28, 31). The infectivity of the assembled HDV virions was examined using *in vitro* infection of HepG2-NTCP cells expressing on their surface functional HBV receptor sodium taurocholate co-transporting polypeptide (NTCP) (2, 37), which was a gift of Dr. Guangxiang Luo. The HepG2-NTCP cells were cultured in 48-well plates on rat tail collagen (Sigma) using advanced DMEM/F12 (Gibco) medium that was supplemented with 10% fetal bovine serum (FBS), 4 mM L-glutamine, 2 μg/mL puromycin, and 1× antibiotic/antimycotic solution. The infection medium, which consisted of DMEM (Gibco) medium that was supplemented with 5 μg/mL hydrocortisone solution, 1% DMSO, and 1× antibiotic/antimycotic solution, and was bearing HDV and 5% PEG, was incubated with HepG2-NTCP cells overnight. After the overnight incubation, the infection medium was removed and replaced with DMEM (Gibco) medium that was supplemented with 10% FBS, 2 mM L-glutamine, 5 μg/mL hydrocortisone solution, 1% DMSO, and 1× antibiotic/antimycotic solution, and which did not contain HDV or PEG. For the analysis of the accumulation of the infection-generated HDV G RNA, the cells were infected using the multiplicity of infection of 70 (i.e., the MOI was 70 HDV GE/cell). For the analysis of HDV-infected cells by immunofluorescence, the infection was conducted using the MOI of 100. The infected cells were harvested at day 8 post-infection, and then either used for the isolation of total RNA using TRI reagent or were further used for immunofluorescence.

### Analysis of HDV-positive infected HepG2-NTCP cells by immunofluorescence

The HepG2-NTCP cells were fixed using 4% paraformaldehyde, then washed twice with PBS and permeabilized using 0.2% Triton X-100. The rabbit polyclonal antibodies raised against the peptide, whose sequence EGGSRGAPGGGFVPNLQGVPESPFSRTGE is present on the delta antigen (HDV genotype 1, M21012) (GenScript) and was used previously by others for the generation of antibodies against delta antigen (53), were employed for staining for delta antigens (both small and large forms) in order to identify HDV-infected HepG2-NTCP cells. For staining of human alpha-tubulin in HepG2-NTCP cells, we used mouse monoclonal antibodies (Sigma). DAPI (4′,6′-diamidino-2-phenylindole) was used to stain for nuclear DNA. Stained HepG2-NTCP cells were then analyzed using an inverted Nikon TE2000-U microscope with a 20× objective. Using staining for alpha-tubulin and for nuclear DNA, the total number of cells was quantified. Using staining for delta antigens, the number of HDV-infected cells was quantified. The fraction of HDV-infected cells was calculated by dividing the number of delta antigen-positive cells by the total number of cells.

### Analysis of the levels of secreted HBsAg by ELISA

For the samples shown in Fig. 3, the levels of secreted HBsAg in the media from transfected Huh7 cells were assayed using HBsAg-specific ELISA (Abbexa).
